# Understanding antimicrobial use by equine owners in Wales: Using cross‐sectional survey and semi‐structured interviews

**DOI:** 10.1111/evj.14522

**Published:** 2025-05-20

**Authors:** Rebekah B. Stuart, Fleur Miles‐Farrier, Alison M. Bard, Gwen Rees

**Affiliations:** ^1^ School of Veterinary Science Aberystwyth University Penglais Hill UK; ^2^ Bristol Vet School University of Bristol Langford UK; ^3^ Arwain DGC Mentera Aberystwyth UK

**Keywords:** antibiotic resistance, horses, owner behaviour, qualitative

## Abstract

**Background:**

Antimicrobial resistance (AMR) is an increasingly serious threat to human and animal health. Antimicrobial use (AMU) in horses is gathering research interest, although there remain significant evidence gaps. Currently, there is scant qualitative research into equine owners' use of antimicrobials, specifically antibiotics, hindering the design of evidence‐based stewardship interventions and policies.

**Objectives:**

To understand Welsh equine owners' views and behaviours relating to antimicrobial usage.

**Study Design:**

Cross sectional survey and qualitative data collection and analysis.

**Methods:**

An online survey (*n* = 319) and semi‐structured interviews (*n* = 21) were conducted with Welsh equine owners. The survey examined participant knowledge of and relationship with their horse(s), sourcing of antibiotics, owner–vet interactions on antibiotic prescribing, antibiotic usage, and their understanding of AMR. Semi‐structured in‐depth interviews further explored horse owner perspectives, experiences, and decision‐making relating to these areas, to add nuance and depth to quantitative data. Survey data were descriptively analysed, and interview data were coded and qualitatively analysed using a thematic approach.

**Results:**

Welsh equine owners regarded their horses as members of the family (84.8%). Most sourced antibiotics from their vet (68.9%) with a minority (5.1%) never having done so or having used antibiotics left over (16.6%) and 13.6% would consider changing veterinary practice if they did not receive antibiotics when they requested them. Interview data suggest that antibiotic use was shaped by (i) key relationships of care (human–animal and owner–vet); (ii) competing priorities (treatment need versus ease of administration); and (iii) imperfect comprehension (education, information, AMU/AMR knowledge).

**Main Limitations:**

Potential self‐selection bias of respondents due to the voluntary nature of participation.

**Conclusions:**

This study highlights variations in the horse‐owner relationship, conflicting priorities involved in horse ownership, and the quality of vet–client relationships which may influence treatment decision‐making. It raises some concerns about the sourcing of antibiotics and variable understanding of AMR, with important implications for antimicrobial stewardship and educational priorities.

## 1 INTRODUCTION

Antimicrobial resistance (AMR) is an increasingly important threat to human and animal health, driven by antimicrobial usage.^1^ The United Kingdom's 5‐year national action plan^2^ outlines the need to work with the veterinary profession to encourage best practice for infection control in companion animals and horses. Antimicrobial use and AMR in companion animals and equids is receiving increased attention; however, there are still large evidence gaps that need addressing.^3^ Antimicrobial sales figures published in the annual VARSS report^4^ provides some indication of AMU in animals; however, these data are of limited value due to their lack of granularity and inability to discern off‐licence use or use of multi‐species licensed products.^3^ Studies have shown that antimicrobials were prescribed in 11%–19.5% of consultations in equine practice in the United Kingdom,^5^, ^6^ with half of UK equine veterinary surgeons (vets) working in a practice that had an explicit antimicrobial stewardship policy.^3^ Complex interventions aimed at improving veterinary antimicrobial stewardship have been developed and implemented in the United Kingdom, largely aimed at AMU and AMR in farm animals.^7^ In the equine sector, resources have been developed to support practitioners to prescribe responsibly, such as BEVA's Protect‐Me toolkit.^8^

While improvements in the data on antimicrobial use and resistance in the equine sector are still required, it is known that one key area for addressing AMR is to understand the human behavioural aspects of antimicrobial use.^8^, ^9^, ^10^, ^11^ Despite recent research on antimicrobial use and prescribing by equine vets, there remains very little evidence currently available on the way in which equine owners source and use antimicrobials or the drivers and barriers for stewardship in this population. Qualitative research methodologies have been shown to provide rich, deep data that can help researchers and policy makers understand the context and experiences of a population of interest, key to the design of appropriate, evidence‐based interventions and policies.^12^, ^13^

The aim of this study was to explore equine owners' views and behaviours relating to the use of antimicrobials—in this case specifically antibiotics—in Wales, using a cross‐sectional survey and semi‐structured interviews. The survey explored participant knowledge of and relationship with their horse(s), their sourcing of antibiotics, experiences of owner–vet interactions on antibiotic prescribing, approaches to antibiotic use and treatment, and their understanding of antimicrobial resistance. A series of semi‐structured in‐depth interviews further explored horse owner perspectives, experiences, and decision‐making relating to these topic areas.

## 2 MATERIALS AND METHODS

### 2.1 Cross‐sectional survey

#### 2.1.1 Design and data collection

An online survey (Survey S1) was created using JISC software (JISC Online Surveys©, 2020). [Correction added on 29 May 2025, after first online publication: The citation of the Survey was corrected from S2 to S1.] The questionnaire was developed by academics with experience in the equine sector, behavioural science, and antimicrobial stewardship interventions (the authors). The survey (Survey S1) consisted of six main sections: information and consent, participant demographics, information on the main horse(s) in their care, antibiotic usage in their horse(s) and understanding of antimicrobial resistance. Questions were a mixture of closed questions, point Likert scale statements, and open‐ended questions. Prior to launch, the survey was piloted with five horse owners and two members of the wider project team with equine experience to ascertain that the questions were clear and concise, and that valid data would be obtained. No major changes were required following the pilot. The survey was available in both English and Welsh languages.

Survey responses were obtained over a 14‐week period from November 2022 to February 2023. The survey was launched during World Antimicrobial Awareness Week in November 2022 (18–24 November) and disseminated via social media channels online and cascaded through newsletters and contacts within the Arwain DGC project; a collaborative initiative between academia and industry within which this research was situated.^14^ Additional data were collected in person at the Royal Welsh Agricultural Society (RWAS) Winter Fair (28–29 November 2022) and Horses Inside Out Annual Conference (11–12 February 2023), through recruitment of the public to engage with data entry on handheld tablet computers. Following completion of the survey, respondents had the option of agreeing to participate in the semi‐structured interviews by entering their contact details for further recruitment information.

#### 2.1.2 Data analysis

Descriptive analysis was performed on the survey data, which included mean, median, and range for continuous data and the percentage frequencies and mode for nominal data. Participant responses to open‐ended questions were analysed using summative content analysis methodology, as described by Hsieh and Shannon.^15^ Using an inductive approach, repeated key words and phrases were identified, coded, and sorted into main themes. These were decided upon by the process of manifest content analysis,^16^ whereby each open question response was categorised into codes dependent on words/phrases used by the survey participant. Descriptive analysis (i.e., frequency counts) was performed to highlight the most common coded content within these responses.

Not every respondent answered every survey question; therefore, the number of responses reported for each question varied and is stated throughout.

### 2.2 Semi‐structured in‐depth interviews

#### 2.2.1 Interview development and sample

A qualitative semi‐structured interview approach was chosen to allow the interview process to remain flexible to follow interviewee interest, knowledge, and insights relating to the research question. Key interview areas of focus were determined by the research team following engagement with relevant scientific literature, and an interview topic guide (Schedule S2) was developed. The interview structure covered five broad areas: (1) Human–animal relationship and interaction, (2) current horse health care, (3) vet–owner relationship, (4) treatment decision making, and (5) understanding of antimicrobial resistance. Within these five areas, the specifics of the questions and topics of conversation were shaped by the interviewees and their responses to facilitate open discussion.

Four pilot interviews were conducted with horse owners to ensure the topic guide stimulated effective discourse around the topics of interest and produced valid data. Interviewee feedback was sought, and the interviewer asked whether the interviewee had felt that the line of questioning was acceptable and covered all aspects they wished to discuss relating to antibiotic use. From the pilot interviews it was determined that the interview structure was fit for purpose and that no changes were required, with two pilot interviews deemed of sufficient quality (i.e., the interviews allowed for effective conversation and coverage of the interview schedule) to be included in the full dataset.

Remaining interview participants (*n* = 19) were recruited from the sample population of survey participants who agreed to be contacted for interview recruitment (*n* = 102 of total survey participants *n* = 546). Participants were selected and contacted by the primary author (Rebekah B. Stuart) using purposive sampling. Eligibility for the interview was determined as being resident in Wales and owning or being responsible for the care of at least one horse. Participants were selected according to geographical location to ensure a reasonable spread throughout Wales and to also represent a range of experiences and owner types. Interviews were either conducted face‐to‐face in a variety of locations—including participants' homes, equine yards, and places of work (*n* = 6) or remotely by either video‐conferencing software (*n* = 13) or telephone (*n* = 2). All interviews were conducted by the first author (Rebekah B. Stuart) to ensure consistency in approach.

#### 2.2.2 Data analysis

Interview recordings were transcribed and were analysed qualitatively by the first author (Rebekah B. Stuart) to identify recurrent and common responses, consensus, and variance within and between the interviewees. Thematic analysis was chosen as a methodological framework for interview analysis, as it can be applied flexibly, without a priori theoretical assumptions about what may be learned from the data. This process was informed by Braun and Clarke's^17^ iterative thematic analysis steps (data familiarity, generating initial codes, searching for themes, reviewing themes, defining, and naming themes, producing a report).

Transcripts were initially imported and coded by the first author (Rebekah B. Stuart) using NViVo software (QSR Software, NViVo 14) to identify core perspectives and experiences represented within interview data. Coding and transcript data were then examined to identify common, recurring patterns across the dataset that could be identified and clustered around a central organising concept or idea (theme) resonating with the research question. Coding and thematic development were supported by authors Alison M. Bard and Gwen Rees as experienced qualitative researchers, who independently coded a subset of data to compare coding, examine the resonance of themes, and critically review thematic structure. The process of writing was a further analytic and creative process, stimulating critical assessment and adjustment of the thematic content to best represent participant perspectives, experiences, and insights.

## 3 RESULTS

### 3.1 Survey response and demographics

**FIGURE 1:**
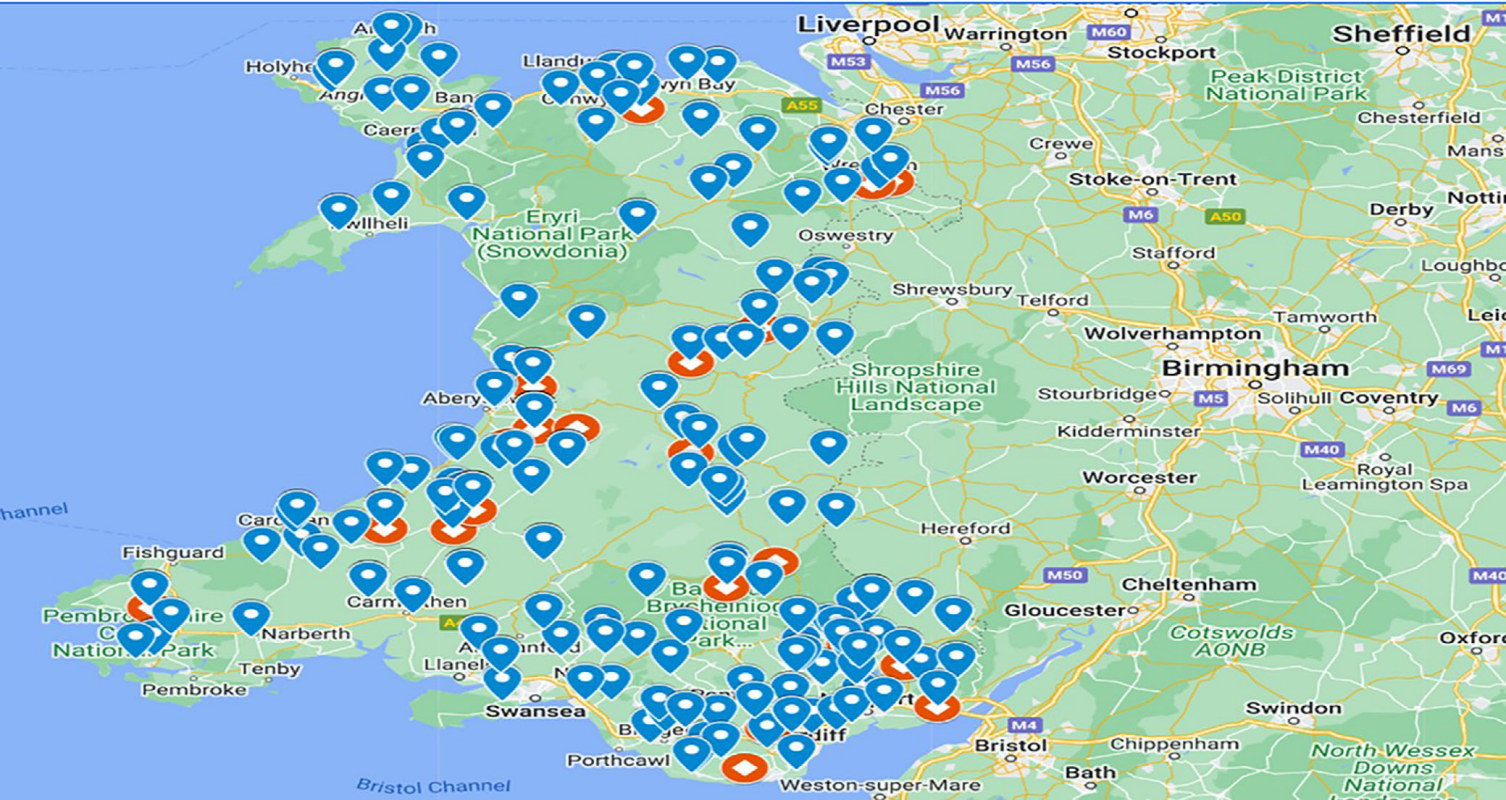
Map highlighting postcode locations of the online survey respondents (*n* = 319, blue diamonds) and interview participants (*n* = 21, red diamonds), codes of postcode districts are written in blue. Some diamonds represent >1 participant and this diagram indicates geographical spread but not specific distribution.

There were completed survey responses, with over 96% of participants from the United Kingdom (529/546). For this paper, only Welsh respondents were included in the analysis as the sample of interest (*n* = 319; Figure 1). As some respondents did not answer every question, the sample size varies throughout the results and is therefore reiterated for each analysis (25 of our survey questions had a response rate >95%, 3 questions had a response rate of 93%). A summary of survey respondents' demographic data and participant experience, knowledge, and relationship with horses can be found in Table 1.

**TABLE 1:** Survey demographic data and participant experience, knowledge and relationship with horses.

Question/Statement	Response	Number of responses	Total number of question respondents	%
Location	UK‐based	529	544	97.2
	Wales‐based	319		58.6
Sex	Female	292	319	91.5
	Male	21		6.6
	Other/Prefer not to say	6		1.9
Age	18–24	24	316	7.6
	25–34	35		11.1
	35–44	57		18.0
	45–54	55		17.4
	55–64	101		32.0
	>65	44		13.9
Number of horses you are currently responsible for	0	4	318	1.3
	1–5	239		75.2
	6–10	33		10.4
	11–20	21		6.6
	>20	21		6.6
Number of horses you have previously been responsible for	0	13	318	4.3
	1–5	108		35.8
	6–10	79		26.2
	11–20	50		16.6
	>20	52		17.2
Number of hours spent with horse(s) each week	<5	30	319	9.4
	6–15	109		34.2
	16–35	119		37.3
	>35	61		19.1
Years of experience with horses	0–10	19	319	6.0
	11–20	39		12.2
	21–30	46		14.4
	>30	215		67.4
Level of knowledge about horses and horse‐care	None	1	318	0.3
	Very little	1		0.3
	Some	29		9.1
	Good	202		63.5
	Excellent	85		26.7
I consider my horse a working animal	Strongly disagree	46	307	15.0
	Disagree	67		21.8
	Neutral	76		24.8
	Agree	90		29.3
	Strongly agree	28		9.1
I consider horses are livestock	Strongly disagree	41	307	13.4
	Disagree	65		21.2
	Neutral	77		25.1
	Agree	98		31.9
	Strongly agree	26		8.5
I consider my horse a pet	Strongly disagree	8	308	2.6
	Disagree	32		10.4
	Neutral	54		17.5
	Agree	126		40.9
	Strongly agree	88		28.6
I consider my horse is a part of the family	Strongly disagree	8	316	2.5
	Disagree	7		2.2
	Neutral	33		10.4
	Agree	110		34.8
	Strongly agree	158		50

#### 3.1.1 Sourcing of antibiotics

**FIGURE 2:**
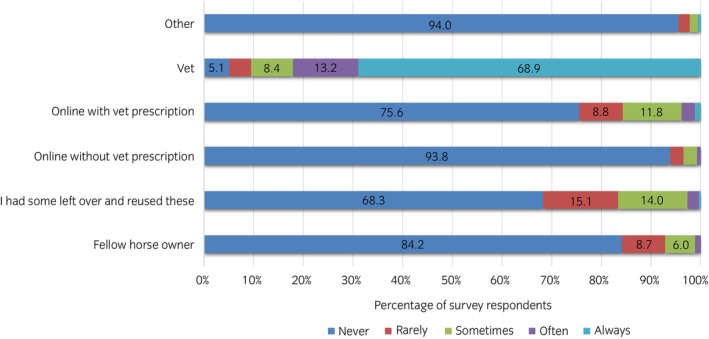
The percentage (%) of responses to ‘Where do you source your antibiotic treatments from?’ Response rates of <5% are not labelled.

Figure 2 presents how participants ranked statements regarding sourcing of antibiotics, with 39% (*n* = 126/319) of respondents reporting the use of multiple antibiotic sources (i.e., selecting >1 survey option). Most participants sourced antibiotics directly from their vet 68.9%, *n* = 204/296, (95% CI 63.6–74.1), with a minority (5.1%, *n* = 15/296, 95% CI 2.5–7.5) never having done so, or having experience of ‘sourcing online without veterinary prescription’ (6.2%, *n* = 16/259, 95% CI 3.2–9.1). Many respondents (31.7%, *n* = 84/265, 95% CI 26.1–37.3) indicated that they had experience of having ‘some [antibiotics] left over and I reused these’, while 15.8% (*n* = 42/265, 95% CI 11.5–20.2) had sourced antibiotics from fellow horse owners.

#### 3.1.2 Owner–vet interactions on antibiotic prescribing

**FIGURE 3:**
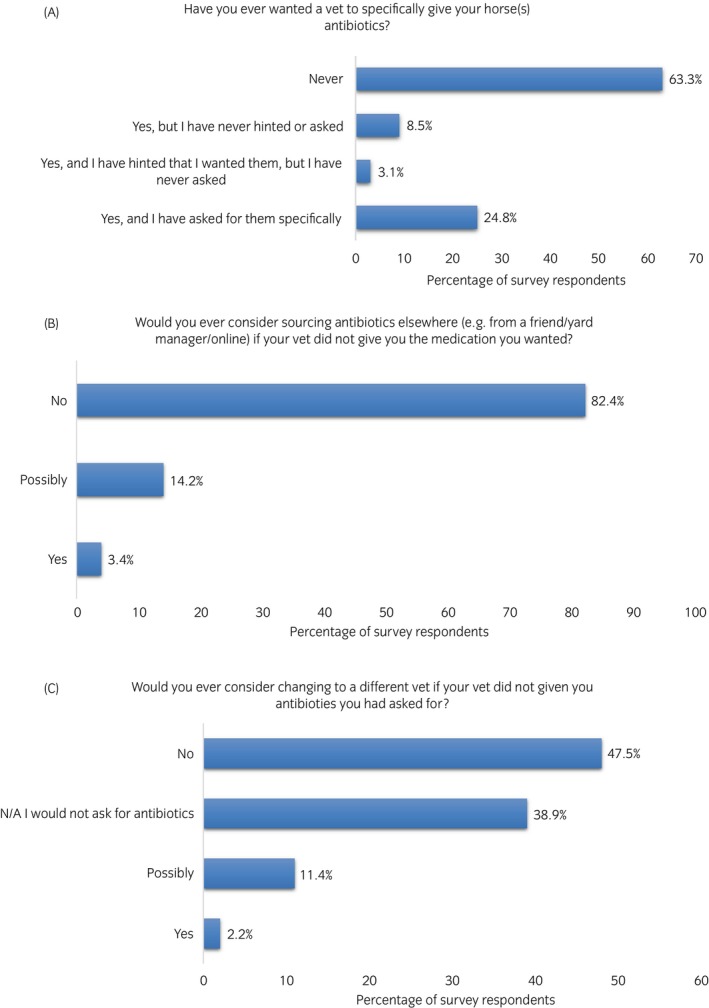
Percentage (%) of survey responses to (A): ‘Have you ever wanted a vet to specifically give your horse(s) antibiotics?’ (B): ‘Would you ever consider sourcing your antibiotics elsewhere?’ (C): ‘Would you ever consider changing to a different vet if your vet did not give you antibiotics you had asked for?’

Nearly a quarter of respondents (24.8%, *n* = 79/318, 95% CI 20.1–29.6) reported that they had specifically asked their vet for antibiotics on occasion, although the majority (63.3%, *n* = 202/318, 95% CI 58.2–68.8) had never done so; 17.6% (*n* = 56/318, 95% CI 13.4–21.8) of participants considered sourcing antibiotics from elsewhere if the vet did not provide them with the medication they wanted, and 13.6% (*n* = 43/316, 95% CI 9.8–17.4) would consider changing vets if their vet did not prescribe the antibiotics they had asked for (Figure 3).

#### 3.1.3 Antibiotic use and treatment

**FIGURE 4:**
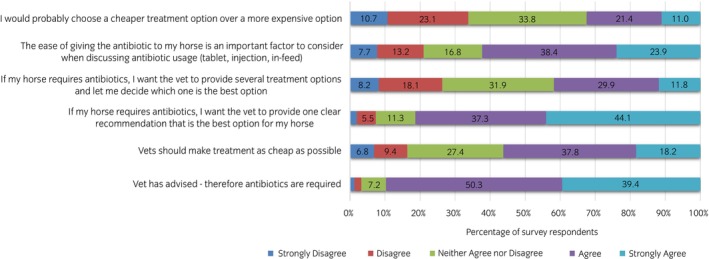
Percentage of responses and the level of agreement with each statement regarding antibiotic usage and treatment for their horse(s). Response rates of <5% are not labelled.

Participants were asked to rank statements relating to antibiotic usage and treatment for their horse(s) (Figure 4). With regards to cost of treatment, just over half of respondents (56%, *n* = 172/306) agreed that vets should make treatment as cheap as possible compared to a minority who disagreed (16.2%, *n* = 50/306), while opinions on selecting a treatment based on cost were evenly split in opinion, between disagreement (33.8%, *n* = 104/308), neutrality (33.8%, *n* = 104/308) and agreement (32.4%, *n* = 100/308) with ‘I would probably choose a cheaper treatment over one that is more expensive’. The majority (62.3%, *n* = 193/310) of participants felt ease of antibiotic treatment is an important factor to consider in prescribing, while agreement with adhering to veterinary advice that an antibiotic is required was extremely high (89.7% agreement, *n* = 280/312). More participants indicated agreement with ‘I want the vet to provide one clear recommendation that is best for my horse’ (81.4%, *n* = 253/311) than ‘I want the vet to provide several treatment options and let me decide’ (41.7%, *n* = 127/304).

#### 3.1.4 Participant understanding of antimicrobial resistance

A total of 80.5% (*n* = 255/318) of the survey participants had heard of antimicrobial resistance (AMR). The survey included a free‐text question where respondents described antibiotic resistance in their own words. From the 149 responses, participants used language relating to both the mechanisms of AMR (i.e., the effectiveness and actions of AMU and the consequences of AMR) and the consequences of AMR (i.e., what AMR may mean for human and animal health and any worries associated with this) (Table 2).

**TABLE 2:** Keywords and themes identified in the responses to the question, ‘What does antimicrobial resistance mean to you?’

Themes	Codes	Example keywords	No. of respondents
Mechanisms	Formal terminology microbes, antimicrobials	Bacteria; bacterial; microbe(s); microbial; pathogen(s); infection (only when the word infection was used without bacteria, that is, bacterial infection). Antibiotics; antimicrobials	213
	Informal terminology microbes, antimicrobials	Organisms; bugs; disease; germs; virus; drugs; medication(s)	71
	Ineffective	Ineffective; no longer effective; no longer works; [bacteria] no longer killed; less useful	90
	Resistance terminology	Antibiotic resistance; [bacteria] resistant to; [develop the means to] defeat; no longer respond; [develop/builds up] immunity; [bacteria] mutate, mutates; [bacteria] adapted; [bacteria] evolve	128
	Misuse	Overuse; overprescribed; misused; incorrectly	86
	Inaccurate	Animal/body/horse becomes resistant; resistant to bacteria; resistance of antibiotics; superbugs not working	19
Consequences	Concern	Concerns, worry, need to protect; future antibiotics [will not work]; future infections [untreatable]; increasing problem; killing; death; [horse will] suffer.	22
	Implications	Finish/complete course [of antibiotics]; only use when necessary; prevention; research; alternative drugs; lack of new antibiotics; responsible use; one health; threat to both human and animal health; [threat to] human health	27

##### Mechanisms of AMR

Almost half of responses chose to discuss that AMR makes antimicrobials ‘ineffective’ (46.3%). Over half of participants (65.8%) chose to use language that directly or indirectly described AMR, including terminology relating to ‘resistance’ (resistance, defeat, no longer respond) (55%), ‘immunity’ (3.4%), and ‘mutate’ (4.7%).

That some microbes (infections) are resistant to frequently used antibiotics, so using that antibiotic is not going to help fight the infection.

When diseases mutate to become resistant to the drugs that are being used to treat them.

Where some form of illness comes immune to an antibiotic/drug.

Some respondents chose to include words or descriptions that suggested a potentially inaccurate understanding of AMR, such as mistakenly describing the animal, horse or body as having resistance (6%, *n* = 9), antibiotics having resistance to bacteria (2.7%) or referring to a virus as the target of antibiotic use (4%):

If you keep having too many antibiotics, your horse is going to get resistant to them and they're not going to work as good as they should be.

It is when the virus becomes immune to the antibiotic and the antibiotic will no longer cure the problem. It is usually caused by overuse or misuse of antibiotics.

##### Consequences of AMR

Few respondents chose to discuss the wider implications of AMR, including AMR being a threat to both human and animal health (4.7%), having only heard of AMR in relation to human health (3.4%, *n* = 5), comparing AMR to anthelmintic resistance in parasitic worms (2%, *n* = 3), and that a course of antibiotics should be completed (2%, *n* = 3).

The same as it means in humans, some bacteria do not respond to antibiotics.

Over use of antibiotics and similar can drive resistance in the same way that worming can drive resistance to anthelmintics.

A minority (8.1%) expressed language relating to concerns and worries regarding a future in which antibiotics do not work, untreatable infections and the potential for suffering.

### 3.2 Semi‐structured in‐depth interviews

Twenty‐one interviews were conducted (Figure 1). The length of interviews ranged from 17 to 125 min (mean = 59.80 min). A description of each of the interviewees, including the role(s) they fulfilled at the time of interview, is provided for each of the participants (Table S1).

**FIGURE 5:**
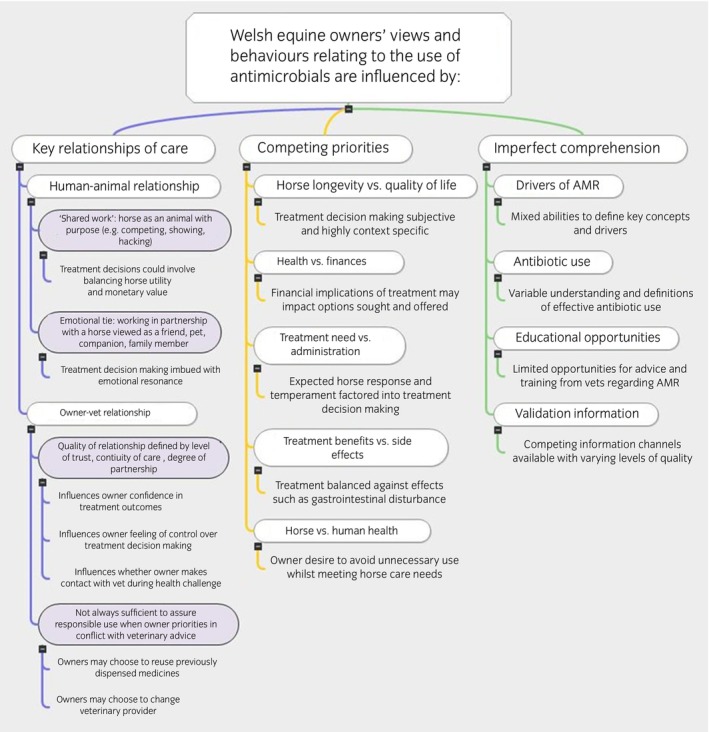
Summary of qualitative thematic results of Welsh equine owners' views and behaviours relating to the use of antimicrobials.

Qualitative thematic analysis of the interview data resulted in three overarching themes regarding the use of antibiotics by equine owners in Wales. Interview data suggest that use is shaped by (i) key relationships of care (both human–animal and owner–vet); (ii) competing priorities; and (iii) imperfect comprehension (Figure 5).

#### 3.2.1 Theme 1 key relationships of care

##### Human–animal relationship

Interviewees described the human–animal relationship as being established both through the demanding nature of both caring for and working with this species. Horse owners described various frequent, time‐intensive activities connected with horse ownership, whether in the process of caring for the animal (e.g., grooming, feeding, stable cleaning, pasture, and yard management) or ‘shared work’ between them (e.g., hacking, competing, showing):

I think doing endurance, actually you get to know your horses much better because you spend so many hours in the saddle. (24)

Combining these experiences with the continuity of long‐term ownership was reported to foster a significant bond, with some interviewees highlighting mutual trust as important. Interviewees frequently referred to their horse as a friend, pet, companion or part of the family that they worked with in partnership, although this was often in tandem with their place as an animal with a purpose:

No definitely part of the family. Part of the family but then I expect them to work as well you know … you know somebody once said to me, a trainer, ‘They don't share our ambitions do they?’ And they don't. You've got to get them to want to do it for you so yes. (15)

This duality in the relationship was reflected in interviewees' perspectives on treatment decision making. When the relational positioning of the horse was as a close emotional tie, this could imbue treatment decision making with the emotional resonance of deciding care for a family member or household pet:

They are the horses that we love. They are part of the family but they are horses. We love them just the same [as our dogs]. We miss them just the same. (27)

When considering the horse's role as an animal of ‘shared work’, treatment decisions could also involve balancing the utility and monetary value of the animal:

I've owned these horses for so long. I do feel emotional responsibility towards them. It wouldn't be any of these higher costly treatments, wouldn't be in the interest of neither of my horses, because [of] the psychological damage to them to be remove from the setting … to like a big clinic where they are being treated for a lot of money … It would be a very different situation if I had a very expensive racehorse or something. (1)

##### Owner–vet relationship

Positive owner–vet relationships were discussed as being built on trust and continuity of care, with many individuals often having built up a rapport with their veterinary practice and/or vets over many years. Interviewees highlighted various qualities as an important part of this relationship, such as being approachable, contactable, and responsive to concerns and worries:

My vets worked very closely with the equine clinic, and they've been fantastic really. Any time I've been worried, they've come to me, and we've worked things out. So yes, I think my relationship with them is very good. (19)

While highlighting the expertise and specialist insight of equine vets, many interviewees also stated a preference for a partnership working model where their vet acknowledged their experience and validated their perspectives in horse health care. This gave interviewees a feeling of control over decision making for their animal and confidence in the outcome:

Do I have an active role? Yes. Do I feel in control? Yes. Do I feel listened to by the vet as well, and they give me the options. I always like to hear all the options, see what the best outcome is for the animal. (1)

The fact that I can speak to them openly and have a discussion, rather than just being told, and the rationale behind why, that to me is the same as if you work with a doctor, it's very, very important…. I need to be confident that what they are telling me is evidence‐based and factual… the vets will obviously know far more than what I do but I need to be confident that it's the right decision for Bob. (28).

This relationship scaffolded interview participants' pursuit of animal health advice and support: within a positive owner–vet relationship, interviewees felt able to easily contact their vet if they chose to seek advice when their horse(s) were showing signs of being unwell, to get a level of reassurance from their vet and to ascertain if a veterinary visit/treatment was required and the level of urgency. Interviewees differed in when this contact was deemed appropriate in the face of health challenge, however, from those who would see it as a first action to those who would see at as the last:

But yes, so yes, my first port of call is always the vet, even if it's only advice. (24)

If I feel the horse is suffering and we cannot provide treatment ourselves, that's when I would call the vet. (1)

Indeed, one interview alluded to the fact that horse owners' providing treatment themselves may do so through using previous prescriptions:

Also, I think for a lot of equine owners they feel they know better and think that actually if they keep some back then they can treat it next time. (28)

Additionally, a positive vet–owner relationship was not always sufficient for owners to remain engaged with veterinary services or treatment decision making as a result of contact. If vets' customer service or horse care was not perceived as sufficient, or when owner care priorities or decision making for their horse care were reported to be different or at odds with their vets', some interviewees reported they may simply choose to pursue treatment options outside this relationship rather than compromise their own treatment desires for their horse(s). This could include changing their veterinary provider:

They were the first ones who came out for [horse]'s first spasmodic colic … he's needle‐phobic and [vet] couldn't get the Buscopan in, so I said, ‘Have you got some Sedalin or Domosedan, preferably, and let's just dose him so that we can get that into the brain?’ [Vet] said, ‘Oh, no, I don't. Sorry’. So, I was like, ‘Hmm, okay, this is a bit of a problem’ [laughs]. So that's why I changed [vets]. (26)

#### 3.2.2 Theme 2: Competing priorities

##### Longevity versus quality of life

Where treatment decisions are concerned, many horse owners were motivated by the quality of life and welfare of the horse(s) in their care. However, various other drivers were identified, including likely treatment outcomes, the potential for animal suffering, and horse age, health, and condition:

I do have a sort of policy if they don't recover from old age. So, if I've got a sort of 30–35‐year‐old horse that needs extensive treatment for something I will pull the plug and say sorry it is time … I don't see the point in just sort of prolonging it if the horse is just going to get worse and worse over time. (20)

As a result, participants described context‐specific decision‐making being very important:

I would make a decision as to whether‐ you know, when do you draw the line on this, as in if we now continue to pump this animal full of something or other, drugs or whatever, are we actually enhancing the value of this animal's life? (10)

With the reality of balancing these subjective values an individual, situated process.

##### Health versus finances

While many interviewees outlined their commitment to ensuring their horse(s)' health, the financial commitments necessary to achieve this were also put forward. Some interviewees reported that veterinary input may involve uncomfortable discussions relating to the financial implications of treatment, and that this may impact upon treatment options sought and offered, and the decision to use or not use antimicrobials. This tension was particularly evident in this interviewee's insight with regards to whether horses were insured:

If you have somebody who is not insured, who has not got a lot of money, who has got a horse who has presented with non‐specific symptoms that could either be bacterial or viral, and the vet said, ‘Right, they may need antibiotics. I need to do a blood test. It's got to go to the laboratory. We're going to check this, this and this’. …. ‘I can't afford that. Can't we just give him the antibiotics in case?’ I think money is going to be the deciding factor. (26)

Perhaps as a result, some owners stated they would only call the vet when they were sure that they could not treat the animals themselves (i.e., signs of colic, wounds, etc.):

Quite honestly, only very, very serious conditions, if it is a very serious part of laminitis and the horse is extremely ill and I think I cannot‐ And they need pain relief, that I can't provide, and I haven't got. (1)

However, for other horse owners, initiating contact with the vet was seen as having no immediate financial consequences, encouraging them to reach out when they are worried as a first option:

Well, there's no point in panicking first of all. I would ring the vet because it doesn't cost anything to talk to somebody. They always say, if you're worried, we're worried. I'd have to tell them what it is I'm worried about it. (21)

##### Treatment need versus ease of administration

Administration ease was sometimes highlighted within the interviews, where a horse's response and temperament in addition to owners' confidence could have a role to play in successful medicine usage:

One of the key ones, ease of use, we didn't realise until with [Bob] that's important to us, so how easy is it to administer it… You know, is there a taste, is there a smell with it? Horses can be very strange… So yes, I would sort of consider is there any implications for refusal because obviously that would impact on whether they successfully complete the treatment. (28)

Although this was not a concern for all interviewees:

I don't have problems administering stuff to the horses at the moment, so I would take vet's advice on what was the best method. (23)

Where an interviewee did report struggling, they noted that if there was a service offered by their veterinary practice to ensure treatment was carried out correctly, they would utilise it:

I do wish that would offer an ambulatory service for a vet nurse to come out and do some things like administering medications. So, that it's done properly, and I would pay for that. (21)

##### Treatment benefits versus side effects

Some owners described their concerns about side effects of antibiotics, particularly gastrointestinal disturbance in the equine microbiota, and the actions they undertook to overcome this:

Especially more so with older and younger ones they can have quite sensitive stomachs and you know I do tend to give a prebiotic then if I am giving antibiotics as well if I am giving antibiotics as well just as a bit of a protective barrier. (8)

One interview participant reflected on her take on antibiotic use in the human context when discussing her horse:

I'm very keen on my gut biome and these having antibiotic just kills it and it won't repair for six weeks. So if there's any other way, any other way, I would rather not have antibiotics which will kill my stomach bacteria thank you. (25)

This interview participant indicated they gave their horses a balancer [feed supplement which can include probiotics targeting gut flora] as a result of concerns for gut health.

##### Horse health versus human health

Some interviewees discussed the need to only use antibiotics when necessary, making the link between horse health and human health and stated a desire to avoid using antibiotics in themselves or their horses. The interviewees' vet was identified as crucial at times of prescribing in being responsible for only prescribing antibiotics when absolutely necessary to meet these intentions:

I think I suppose I trust my vet to be sensible in prescribing what's needed rather‐ you know I don't expect my vet, and I'm sure they wouldn't prescribe something just because they thought I wanted them to, and I think sometimes maybe people want something prescribed just to make them feel better, but I don't. (9)

#### 3.2.3 Theme 3: Imperfect comprehension

##### Drivers of antimicrobial resistance

Interview participants universally expressed an awareness of antimicrobial resistance. Some were able to accurately define key concepts and drivers relating to this term:

This is where bacteria can no longer be killed by antibiotics, that they were previously sensitive to. It's because they're basically, it's like a mutation. It's almost selected ones that are resistant to it, so the antibiotic no longer works against that bacteria, ish. (21)

Although as one interviewee highlighted, this knowledge was not necessarily seen as relevant in consideration of horse care:

I guess I am kind of more aware of it like with the humans, with doctors kind of not wanting to prescribe antibiotics for things like chest infections or throat infections and things like that unless they really need it … But yes, I guess I haven't really thought about it with horses but then I don't suppose you use antibiotics for horses as much as maybe for humans. (17)

Where interviewees had an incomplete understanding of the drivers of AMR and how resistance develops, this could influence behaviour surrounding treatment. For example, one interview participant stated that they would stop/not complete a course of antibiotics if the horse being treated showed signs of improvement given their understanding of ‘overuse’:

Had a course of antibiotics for a week, if she looks better after five days, don't continue with it… I would rather underuse antibiotics than complete a course… [antimicrobial resistance] means getting to the point that whatever medicine is being prescribed may not be effective because there has been a build‐up of over‐usage. (29)

Misunderstandings could be mediated through clear guidance provided by vets that directed treatment action, as described by interviewee 25:

I always ask for clear instructions. I've worked in the care industry so any medication, I want clear instructions please and I usually get it. They're pretty good but you have to ask. I do feel if I hadn't of asked, I wouldn't have got it. (25)

This guidance was valued by this horse owner, although it was not perceived as readily available.

##### Antibiotic use

Interview participants' understanding of and definition of antibiotics varied greatly. Some interviewees could generally differentiate between antibiotics and other medicines (e.g., anthelmintics/wormers, painkillers). Interviewee understanding of where antibiotics had clinical relevance as a treatment option was mixed, with some interviewees specifying their lack of utility in viral infections:

I guess more could be done you know for all livestock, horses, and people, to get across antibiotics don't touch viruses for instance. So you know, I want some penicillin, I've got a cold, it's a bit pointless. (20)

While for others, both bacterial and viral infections were referenced as relevant for antibiotic treatment:

If we don't watch how much we use antibiotics, we're going to have viruses and germs that are resistant to them and we won't have anything to fight the viruses with pretty soon…. I would rather not use antibiotics if there's any other way. In fact we haven't used antibiotics. (25)

For this interviewee, it is not apparent that this misunderstanding necessarily undermined their antibiotic stewardship proclivities, where this conceptualisation still positively contributed to reducing unnecessary use.

##### Educational opportunities

Some interviewees specified conversations around AMR were not a frequent occurrence, referring to a need for wider knowledge transfer and greater engagement from veterinary professionals with the equine sector regarding AMR:

It's the owners, it's about education. Education of the owners to understand what an antibiotic does and what it doesn't do, and actually what potential harm it can do. (21)

##### Validating information

Interviewees reported many competing sources of information available to them in relation to AMR, infectious disease, and the day‐to‐day management of the horses in their care. Several communication channels of information were identified, such as breed societies, social media, publications, and the internet. Some interview participants highlighted their concern with incorrect information being presented on these channels and their difficulty in differentiating between good quality information and misinformation to guide their AMU behaviour:

I think it's difficult these days because people have so many different communication channels. I actually think that Facebook is probably the best one. I have a feeling that's where I found this project was going on, and I think that's probably a really good one. On the other hand, there is loads of misinformation on Facebook, I know that. (24)

## 4 DISCUSSION

This research utilised both quantitative and qualitative approaches to develop an understanding of antibiotic use in the equine sector, studying how Welsh equine owners care for their horses, access veterinary services, and utilise veterinary medicines. To the best of the authors' knowledge, this is the first study to qualitatively explore horse owners' views on antibiotic use in the United Kingdom. The study's focus on Welsh equine owners is pertinent due to the national antimicrobial stewardship programme, Arwain DGC, which has been funding vet–client consultations aimed at reducing antibiotic use in horses in Wales.^14^ The results provide a deeper understanding of horse owners' perspectives, experiences, and decision‐making relating to antimicrobial use in horses.

The results have shown that a considerable proportion of participants consider their horses to be a pet (69.5% of survey respondents) or a member of the family (89% of survey respondents), while perhaps simultaneously viewing them as a working (38.4%) or livestock (40.4%) animal. The human–horse relationship was also identified as an important theme within the semi‐structured interview data, with a similar combination of emotional significance and practical utility, which shaped treatment decision making. These findings are in parallel with results from Clough,^18^ who examined the human–horse relationship regarding decision making at key points within the horse's life. Owners often buy horses for a specific purpose or function, but due to the time spent caring for their horses (e.g., 56% of surveyed owners spent a minimum of 16 hours a week with their horses), they become a valued member of the family. Given that there is extensive research showing that parents will treat children with antimicrobials in a risk‐averse way due to the emotional/relational context of this decision making—that is, they may use antimicrobials ‘just in case’ when possible, even if not necessary^19^, ^20^ with the same inclination shown in companion animal owners^21^ this has implications for the way equine owners might behave when treating their horses. Results from this study additionally identified that horse owners felt they had a ‘duty of care’ to safeguard the wellbeing of their horses, this is reflected in the competing priorities that the interviewees referenced in discussion of treatment decision making. Similar findings have been reported in studies within the agricultural sector.^22^

In human health, inappropriate use of antibiotics by self‐medicating is an increasingly recognised problem.^23^, ^24^ A large EU study highlighted that 46% (*n* = 95/206) of individuals surveyed self‐medicated with leftover antibiotics from a previous prescription.^25^ Equine survey data suggest this ‘self‐treating’ could also be a common practice within the equine sector, with 31.7% of respondents selecting ‘I had some left over and I reused’ as a means of sourcing antibiotics and 15.8% of survey respondents selecting ‘fellow horse owners’ as a route to obtaining antibiotics. This practice was also alluded to within qualitative interviews. This may indicate that antibiotic misuse in the equine sector needs addressing, and certainly deserves further exploration. Where both interview and survey data suggest imperfect knowledge of AMR and AMU are likely to exist in this study population, education and knowledge exchange with horse owners may be a means to achieve positive engagement with ideas surrounding more responsible AMU. Ensuring treatment courses are completed as advised by vets and any leftover/unused antibiotics are disposed of appropriately is crucial to safeguard antibiotics for the future.

Ease of administration was another factor highlighted in this study as important to the use of antibiotics by horse owners in both the survey and interview data, with interviews detailing how administration of antibiotics could be difficult for some owners, depending on the horses' temperament and owner's ability. Research suggests that the method of administration (tablet vs. paste) can impact a horse's stress response, and it follows that some owners find particular medicines easier to administer than others.^26^ Perceived ease of administration—rather than treatment appropriateness with regards to responsible AMU—could therefore dictate the antibiotic choice made by equine vets for some clients as not all antibiotics come in easily administered preparations,^26^ which a vet may anticipate will result in an increased risk of non‐compliance, under‐dosing, and reduced course lengths. Indeed, in small animal practice vets report prescribing feline patients the long‐acting injectable cefovecin (a third‐generation cephalosporin listed as Category B ‘highest priority critically important’ by the European Medicines Agency) as a first‐line antimicrobial agent when a narrow‐spectrum oral antibiotic would suffice, given fears for owner compliance with the latter treatment; of concern, is that these prescribing decisions often underestimate owners' actual capabilities and confidence.^27^ While equine vets may benefit from assessing and incorporating ease of administration in prescribing and formulation choices, this process must therefore also involve careful consideration of any assumptions made regarding the capabilities of their equine clients, and active communication to explore their perspectives.

Another key relationship that impacts the way antibiotics are sourced and used in horses in Wales is the relationship between the equine owner and their vet. The vet–client relationship was an important subtheme within Relationships of Care and was also emphasised within the survey data, mirroring previous publications highlighting the importance of the vet–client relationship.^28^, ^29^ However, qualitative interviews suggested this relationship was not always sufficient for guaranteeing owner engagement with veterinary services or with a recommended treatment approach; owners may instead choose to pursue alternative care options rather than compromise their own treatment desires for their horse(s). Accordingly, while 68.9% of survey respondents identified their vet as ‘always’ the source for antibiotics, 5.1% reported the vet was never the source. Given that antibiotics are veterinary prescription‐only (POM‐V) medicines in the United Kingdom^30^—meaning equine owners can only legally source them from a vet who has the animal under their care—it would therefore appear that there is some evidence of illegal sourcing of antibiotics from other avenues, which is something that requires further research and exploration to understand whether medicines are used legally and responsibly in this sector.

Another important result to note is that over 13% of survey respondents indicated they would consider changing veterinary practice if their vet had not prescribed the antibiotics they had asked for, mirroring similar findings from qualitative studies of pet owners.^21^, ^31^ Similarly, within the interview data, individuals described how they changed veterinary practice on occasions when their veterinary practice could not offer certain services, when owners were left disappointed by the level of care offered by their vet or when their treatment desires were not met by their veterinarian. This suggests that vets may be placed under pressure to prescribe antibiotics against their clinical judgement—whether by financial implications and/or to maintain a positive vet–client relationship—and highlights the difficult nature of navigating responsible prescribing in private veterinary practice. This tension in the relationship between prescriber and recipient is also seen within human healthcare; tension between GPs and their patients when antibiotics were not prescribed was reported by Van Der Zande.^32^ Similar pressures have been noted by vets working within the companion animal sector.^31^, ^33^, ^34^

Participating horse owners' understanding of AMR varied significantly, both in survey data and interview data. Findings from the survey highlighted that over 19% of equine owners had not heard of AMR, while those that had indicated varying levels of accuracy in their understanding. Within the interviews, all participants reported that they had previously heard about AMR, yet, as highlighted in the theme of ‘Imperfect comprehension’ interviewees faced various challenges navigating insight and understanding of this topic. These findings are similar to a review by McCullough et al.^35^ which emphasised that the general public have heard of AMR; however, their understanding was often incomplete, and they felt they did not play a role in AMR development.^31^ Within the pet sector, a lack of awareness of AMR and interspecies transmission has been demonstrated.^31^ This study suggests that there is scope for improvement in understanding of AMR risks and transmission amongst the equine sector in Wales. Focused communication of key messages regarding appropriate use of antibiotics in horses and the risk of AMR, in addition to evidence‐based interventions aiming to engage and promote horse owner motivation regarding responsible AMU, is warranted both at a public health level and specifically within the equine veterinary profession.

### 4.1 Study limitations

With regards to study construction and analysis, this research methodology was designed to enable triangulation of the broad data from the cross‐sectional survey with the rich data obtained by in‐depth interviews. Due to the time limitations of the research, data collection and analysis for both survey and interviews occurred in tandem. Given the identification of practices within survey data—such as self‐treating and re‐use—that are significant in the consideration of responsible AMU, further qualitative study expanding on our interview schedule to include these topics would benefit our understanding of this research context. This study is descriptive, and as such, no inferences can be drawn as to causality or factors associated with antimicrobial use behaviours.

In both these studies, it is important to consider that equine owners participating were self‐selecting through engaging autonomously. This may mean equine owners and carers that were more comfortable with—or interested in—discussing aspects of their relationships with horses, treatment decision making, and AMU/AMR participated. This can also be seen within the results from the survey questionnaire, as a lower response rate was noted for the question regarding ‘sourcing of antibiotics’. It is possible participants did not want to answer the question, felt uncomfortable about how the question was worded, or after answering that they always receive antibiotics from their vet did not feel they needed to respond to other statements.

It is possible that in some instances participant responses have been based on a misunderstanding of which medicines are antibiotics, which would have implications for some of the study conclusions. Additionally, participant recall may have influenced survey and interview responses, as where participants are required to recall events or experiences from the past information collected may include inaccuracies. However, given the need for consent in study participation and personal reflection being the source of survey/interview data, these considerations are intertwined with all survey and interview methodologies.

The generalisability of survey data to the broader population of horse owners and carers within Wales must be considered. A large sample (*n* = 319) suggests reasonable diversity of Welsh horse owner views within the data set, yet given the dearth of specific data on the Welsh horse owner population, it is not possible to determine with accuracy the relationship of these data to national horse owner demographics. At a UK level, 88% of riders are female, as highlighted in a recent survey^36^ suggesting the gender representation in this survey is reasonable at 91.5% female. Given the distribution of the survey via the internet and at in‐person events, populations with reduced access to engagement (e.g., those with reduced internet access, those uninterested in or unable to attend events due to travel, finance or mobility) may be underrepresented in these data. Further granularity of demographic data such as horse owner ‘type’ (e.g., competition horse owner, pleasure horse owner) was not collected in the survey; therefore, it is not possible to ascertain how representative data are for the general population in this regard nor examine the relationship of these characteristics with the antimicrobial use behaviours queried.

With regards to interview data, while the qualitative interview approach offers detailed, valid, and rigorous insight into this research context, it does not seek to quantify opinions within a select group or generate a representative sample of those opinions. As a methodology it can, however, explore and uncover the complexity of horse owner experiences relating to this topic, providing complementary and detailed insight alongside survey data.

## 5 CONCLUSIONS

Study data suggest equine owners care about their horses and are motivated to provide high levels of care, albeit within the constraints of their capabilities and finances. They report using medicines in appropriate ways under vet supervision and that they place value in the advice and services of their vets. At the same time, equine owners in Wales also report self‐treating, storing, and reusing antibiotics and acquiring antibiotics from friends or acquaintances without prescription. While participating owners expressed the importance of their relationship and trust in their vet, it is of note that equine owners stated that they would be willing to change vet to receive the service they expected, which may influence veterinary prescribing behaviour. Equine owners who took part in this study displayed varying levels of understanding of AMR and AMU, identifying a need to improve messaging, education, and engagement with this cohort. While equine owners in Wales are largely using medicines appropriately, further improvements could be made by focusing on the identified issues such as self‐treating and re‐use of medicines, which warrant further investigation through both quantitative and qualitative research. The current findings point to the importance of effective communication when discussing antimicrobial use, particularly in validating veterinary assumptions of client motivation and compliance regarding prescribing and formulation choices.

## FUNDING INFORMATION

The research was funded through the Welsh Government Rural Communities—Rural Development Programme 2014–2020, which is funded by the European Agricultural Fund for Rural Development and the Welsh Government.

## CONFLICT OF INTEREST STATEMENT

The authors declare no conflicts of interest.

## AUTHOR CONTRIBUTIONS

**Rebekah B. Stuart:** Writing – review and editing; writing – original draft; project administration; investigation; conceptualization; data curation; formal analysis; methodology; resources. **Fleur Miles‐Farrier:** Writing – review and editing; project administration; resources. **Alison M. Bard:** Writing – review and editing; conceptualization; formal analysis; methodology; supervision; validation. **Gwen Rees:** Funding acquisition; writing – review and editing; conceptualization; formal analysis; supervision; validation.

## DATA INTEGRITY STATEMENT

Rebekah B. Stuart had full access to all the data in the study and takes responsibility for the integrity of the data and the accuracy of data analysis.

## ETHICAL ANIMAL RESEARCH

This research was approved by Aberystwyth University's Ethics Committee (reference number: 18404).

## INFORMED CONSENT

Participants gave written consent for inclusion in the study.

## Supporting information


**Data S1.** Survey S1: Equine survey.

**Data S2.** Schedule S2: Interview schedule.

**Table S1.** Interview participant information including roles they fulfilled.

## Data Availability

The data that support the findings of this study are available upon reasonable request from the corresponding author. Open data sharing is not applicable to this article due to the qualitative nature of the data in this study.
