# Supplementary material for: Understanding antimicrobial use by equine owners in Wales: Using cross‐sectional survey and semi‐structured interviews
Source: Equine Vet J. 2025 May 20;58(2):564–79. doi: 10.1111/evj.14522 (PMC12892391; doi:10.1111/evj.14522)

Equine Survey

Understanding horse owner perceptions of medicines use and horse health care.

# **
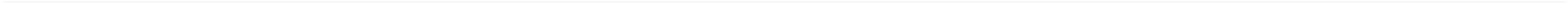
About you**


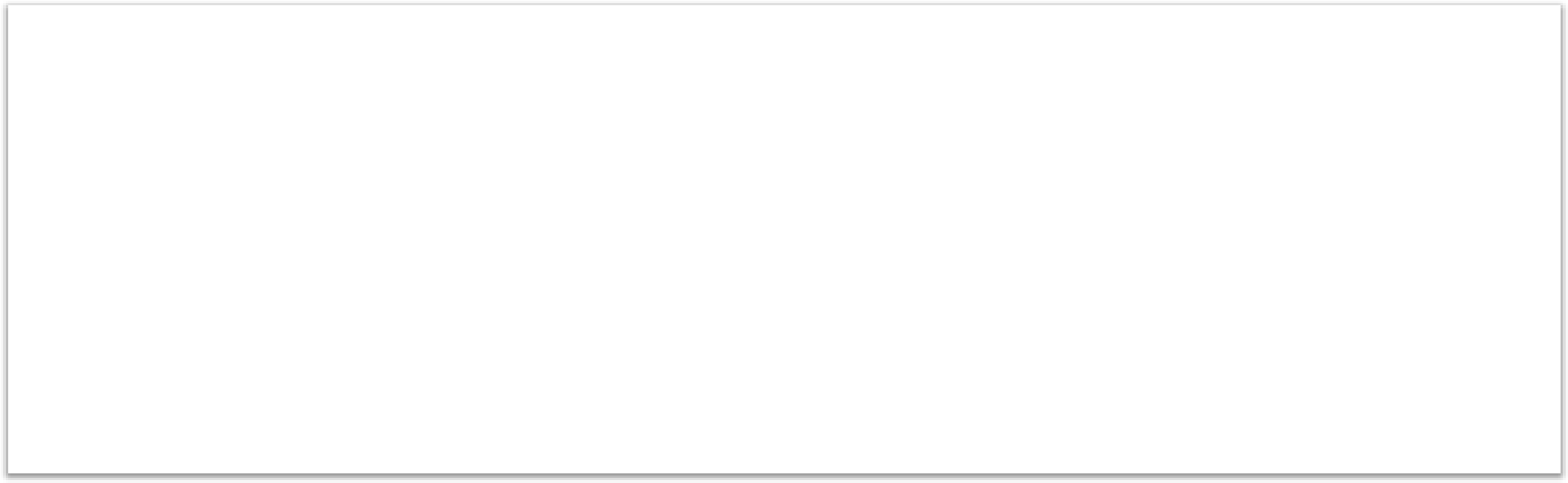


Male

Female

Trans-gender

Non-binary

Prefer not to answer

Other

**To which gender do you most identify?**

If you selected Other, please specify:


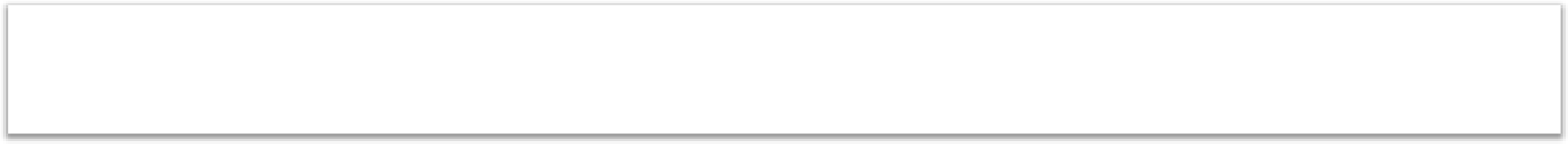

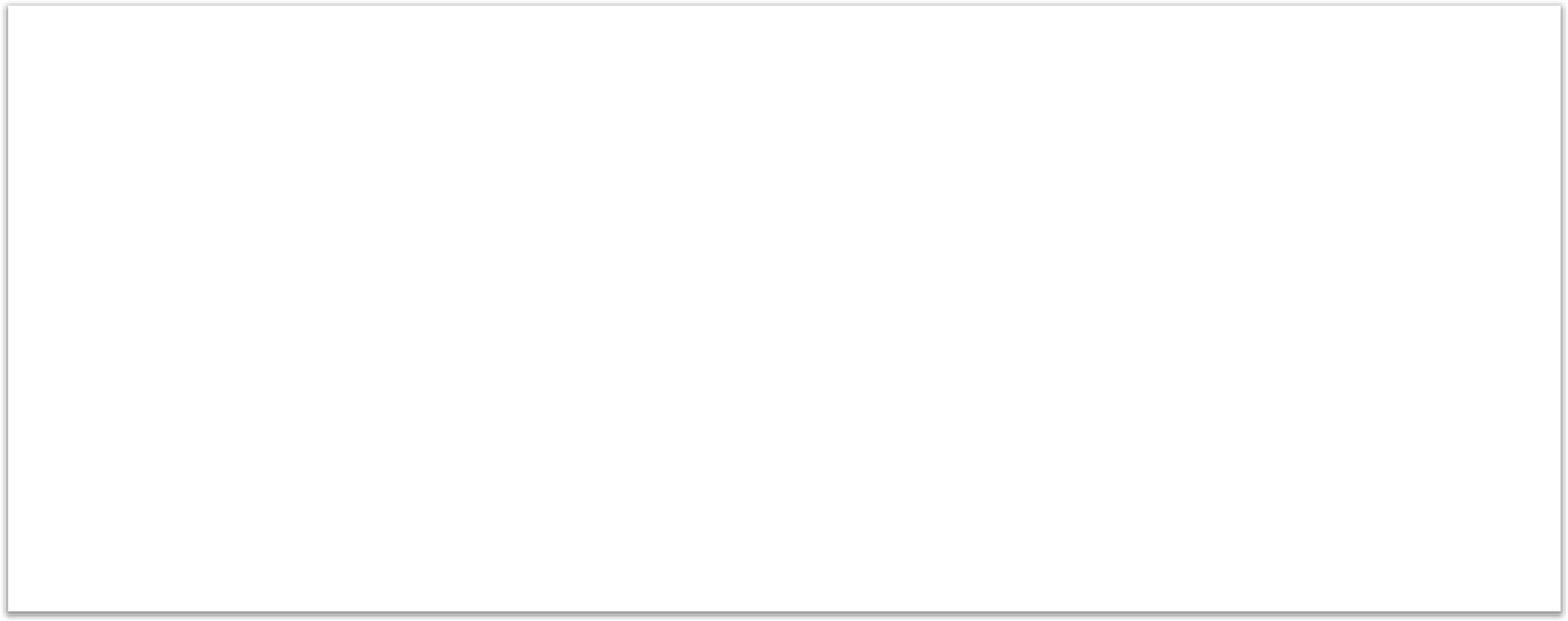


24

--

18

25

-

34

35

-

44

45

-

54

55

-

64

65

-

74

75

+

Prefer not to answer

**What is your age?**

**Postcode?** Please provide us with the first 4 characters of your postcode for your home address (e.g.SY23) this will enable us to identify the region you are located in. (If you live outside the UK please state the country that you live in).


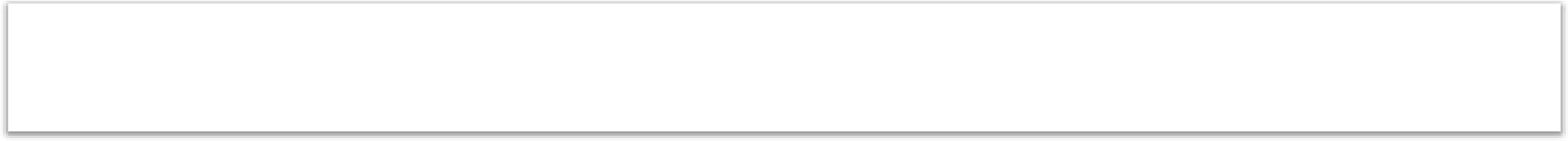

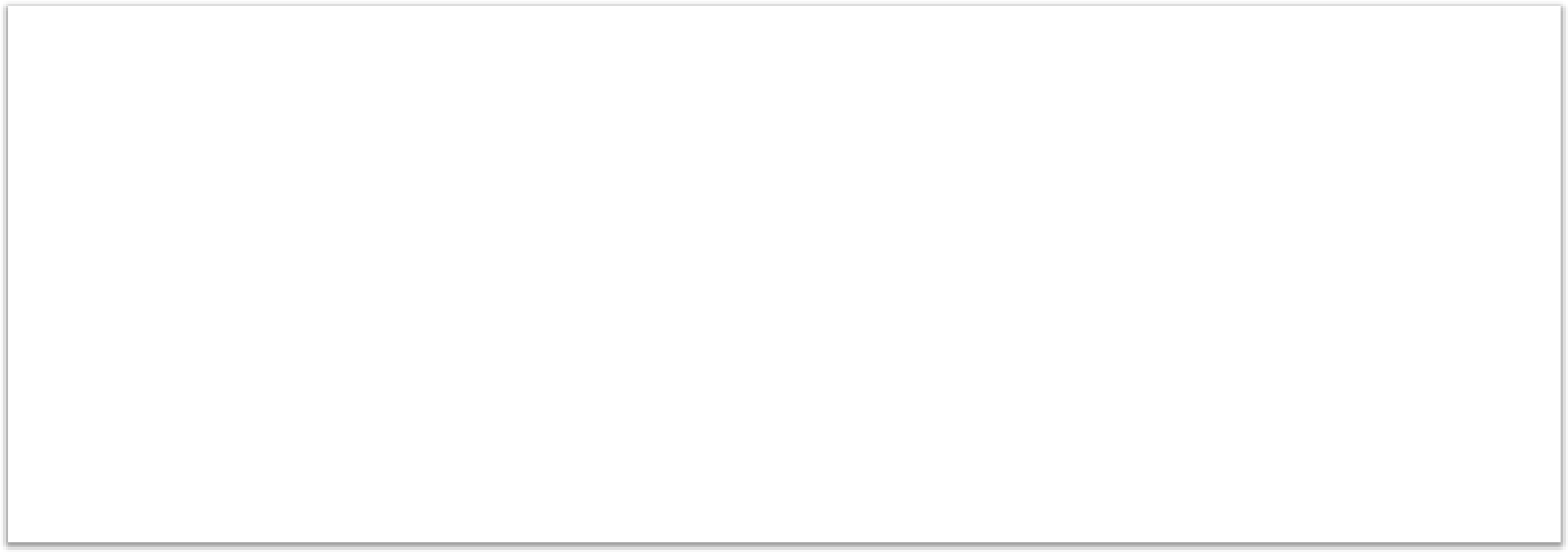


University qualification (undergraduate or postgraduate level)

College courses

Coaching certification

BHS stage exams

Pony club tests

No formal equine education

Other

**Do you have any formal equine education?**

(Please tick all that apply)..

If you selected Other, please specify:


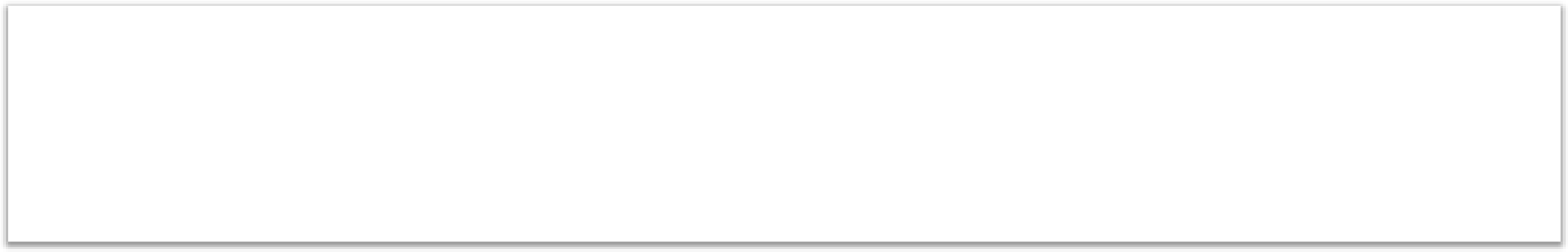


**Have you participated in any Arwain DGC training, activities or events? (E.g. equine health clinic, online webinar)**


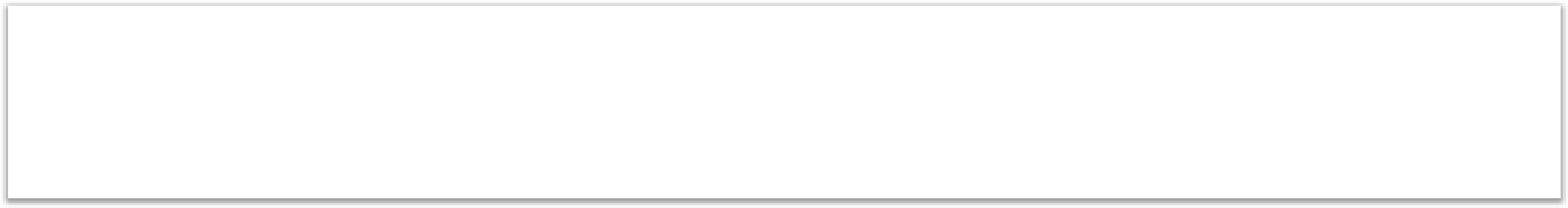


Yes

No

Please describe


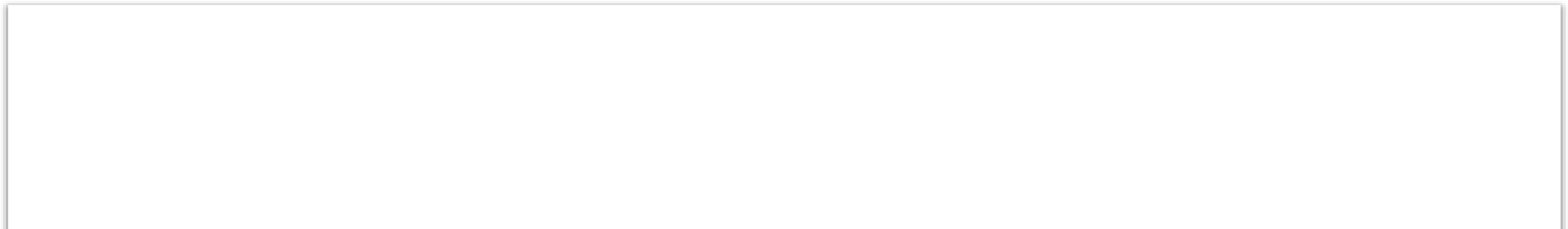

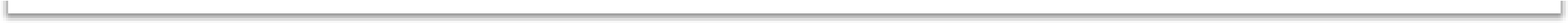


**Are you affiliated with or a member of any equine societies?** (Please tick all that apply).


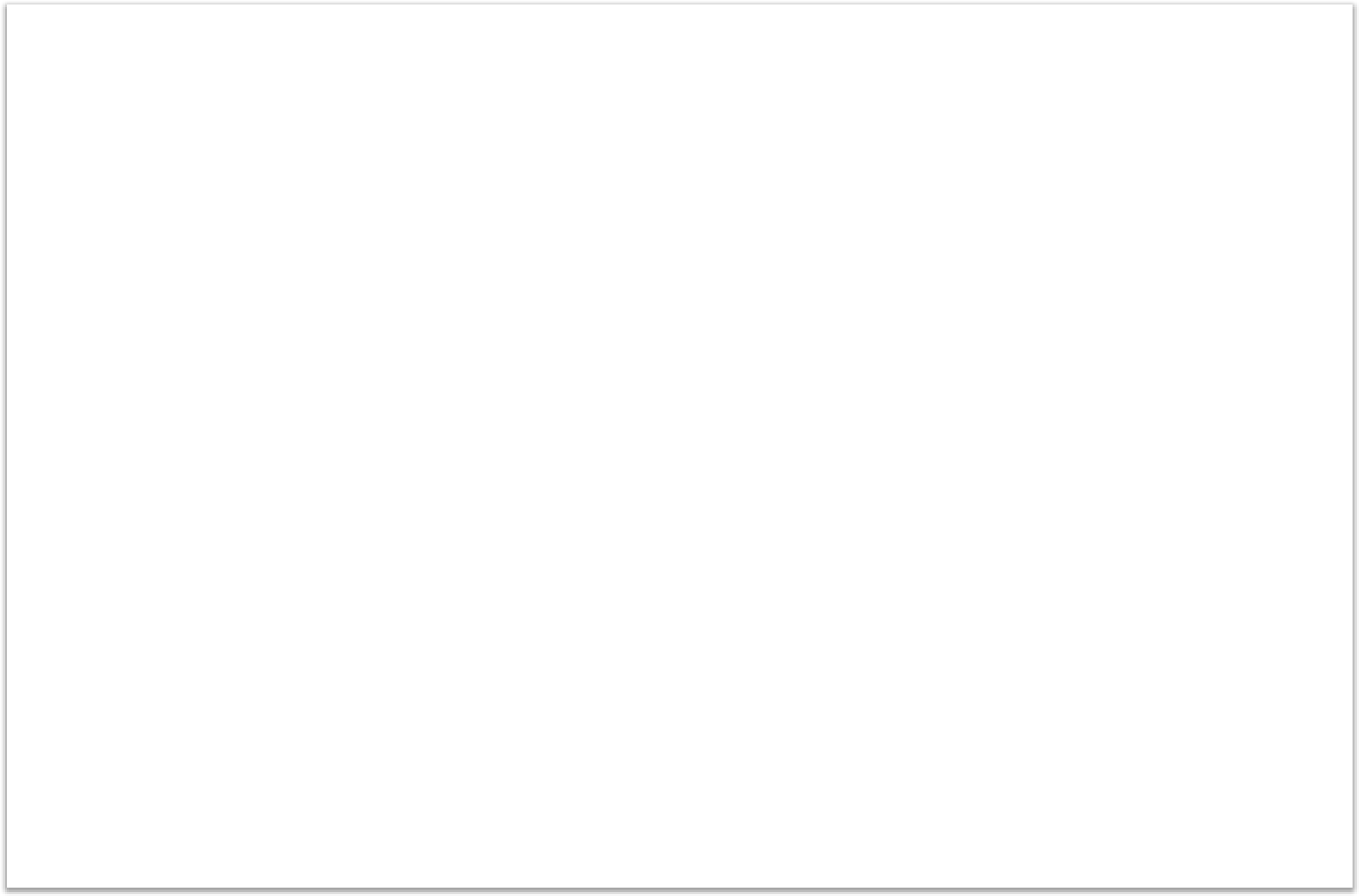


British Horse Society

The Welsh Pony and Cob Society

A Breed Society

British Dressage

British Eventing

British Show Jumping Association

British Show Horse Association

British Driving Society

Veteran Horse Society

A Riding Club

Pony Club

British Groom Association

No affiliation

Other

If you selected Other, please specify:


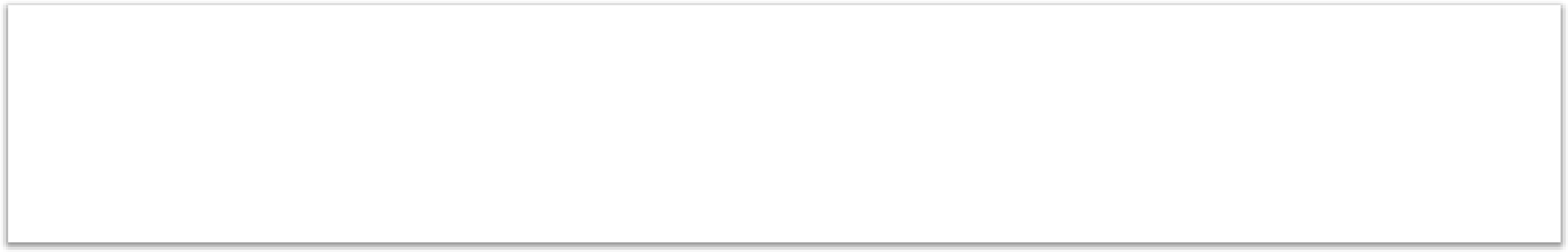


**Please specify the relationship you have to the horse(s) in your care.** (Please tick all that apply).


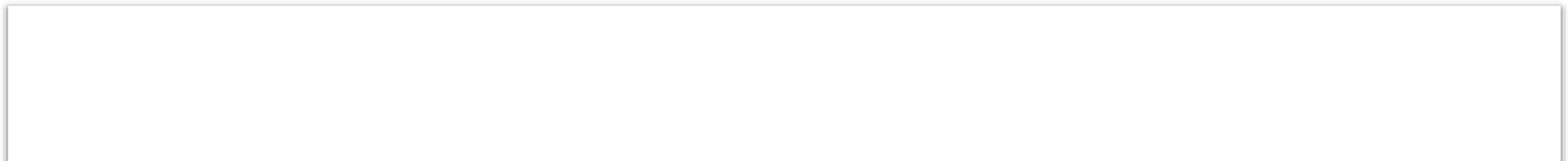


Owner

Trainer


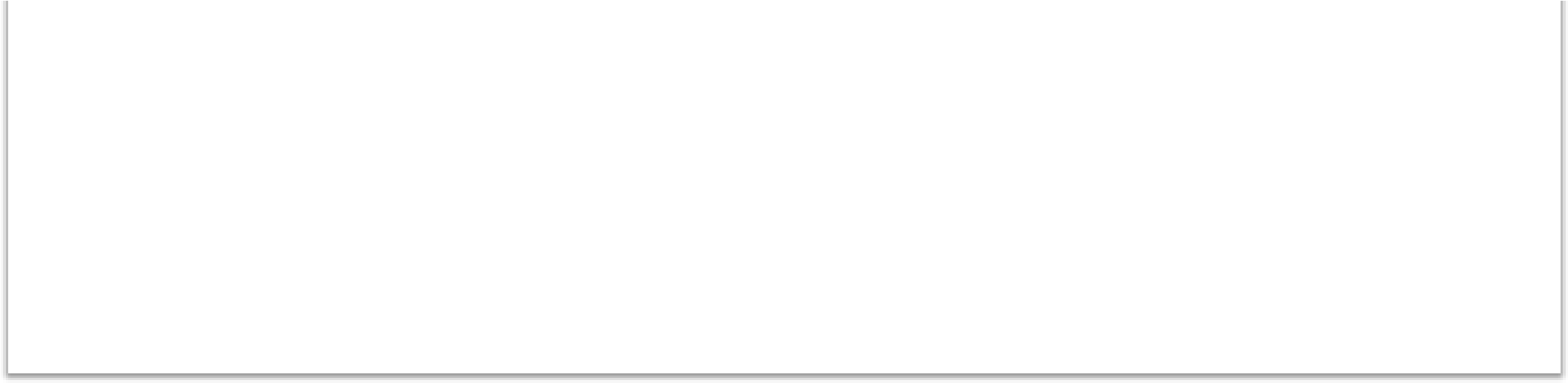


Loan/Share

Yard manager/owner

Groom

Rider

Other

If you selected Other, please specify:


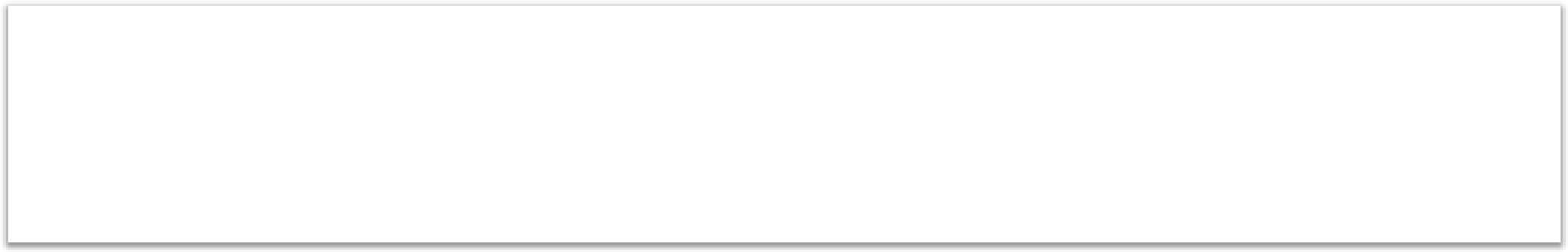


**Please state the number of horses you are currently responsible for.​**


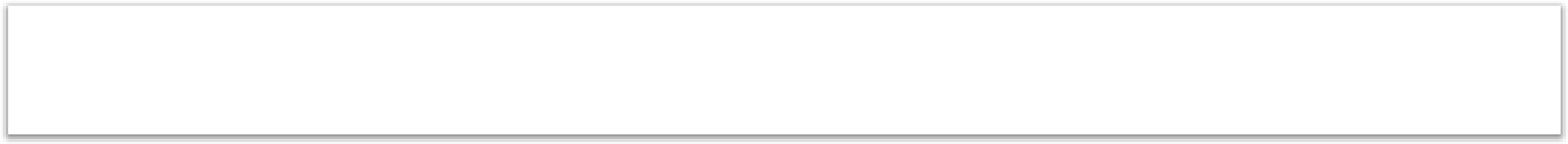


**Please state the number of horses you have previously kept/owned.**


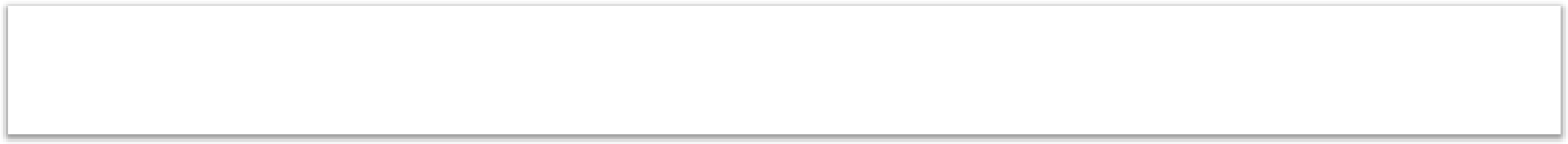


# **Horse-Human Relationship**

**Please indicate along the scale how you view the relationship with your** **horse(s).**

Please don't select more than 1 answer(s) per row.

|  | Strongly Disagree | Disagree | Neither  Agree nor  Disagree | Agree | Strongly  Agree |
| --- | --- | --- | --- | --- | --- |
| Horses are livestock |  |  |  |  |  |
| I consider my horse(s) to be a pet |  |  |  |  |  |
| Working with horses is my profession |  |  |  |  |  |
| Horses are a hobby/passion of mine |  |  |  |  |  |
| I consider my horse(s) to be a working animal (eg for breeding, competition etc) |  |  |  |  |  |
| I consider my horse(s) to be part of my  family |  |  |  |  |  |


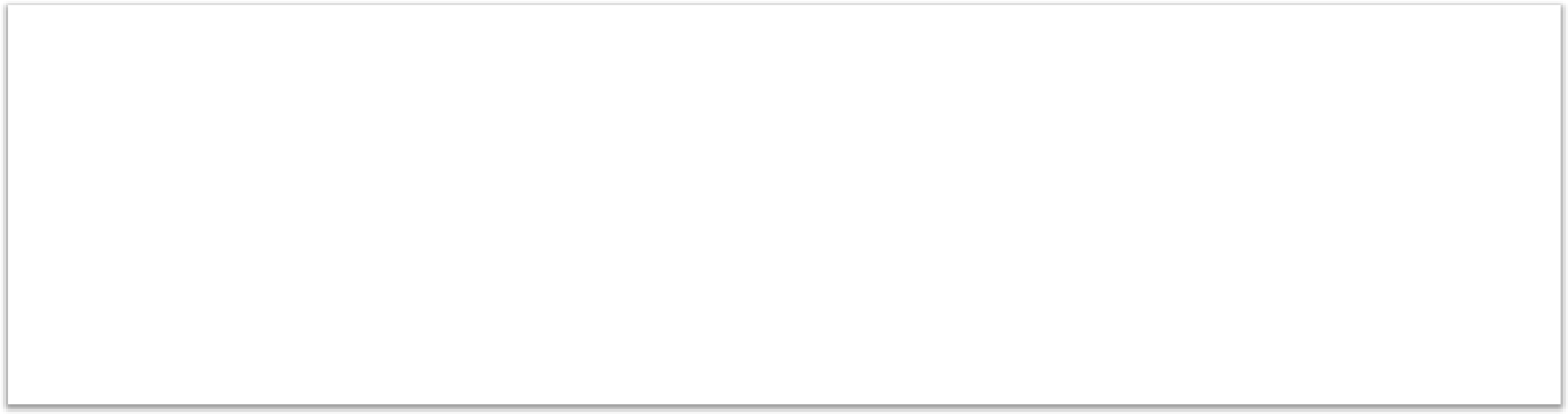


0

-5 years

6

-10 years

11

-20 years

21

-30 years

30

Years or more

**How many years have you been involved with the equine sector?**

**How knowledgeable do you feel about horses/horse care?**

Please don't select more than 1 answer(s) per row.

|  | No knowledge | Very little  knowledge | Some  knowledge | Good knowledge | Excellent knowledge |
| --- | --- | --- | --- | --- | --- |
| Level of knowledge |  |  |  |  |  |


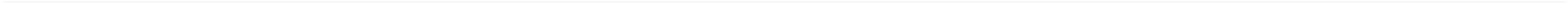
**Please provide us with the first 4 characters of your postcode for the address in which your horse or horses are kept.** (e.g. SY23). You can enter more than one location.


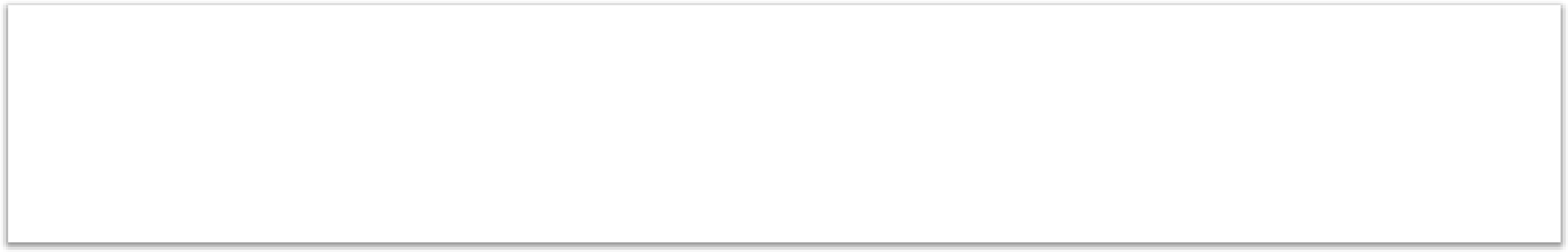


If you live outside of the UK, please state the country that you live in.

**Who provides day to day care for your horse or horses?**

Please rank this list in order of responsibility–from (1) most involved in care; (2) second most involved in care; (3) third most involved in care etc. Please select N/a for any that do not apply to your horse care arrangements.

Please don't select more than 1 answer(s) per row.

|  | 1 | 2 | 3 | 4 | 5 | N/a |
| --- | --- | --- | --- | --- | --- | --- |
| Yourself |  |  |  |  |  |  |
| Family members |  |  |  |  |  |  |
| Friend/other horse owner |  |  |  |  |  |  |
| Paid help |  |  |  |  |  |  |
| Livery yard staff |  |  |  |  |  |  |
| Other |  |  |  |  |  |  |

If other, please describe.


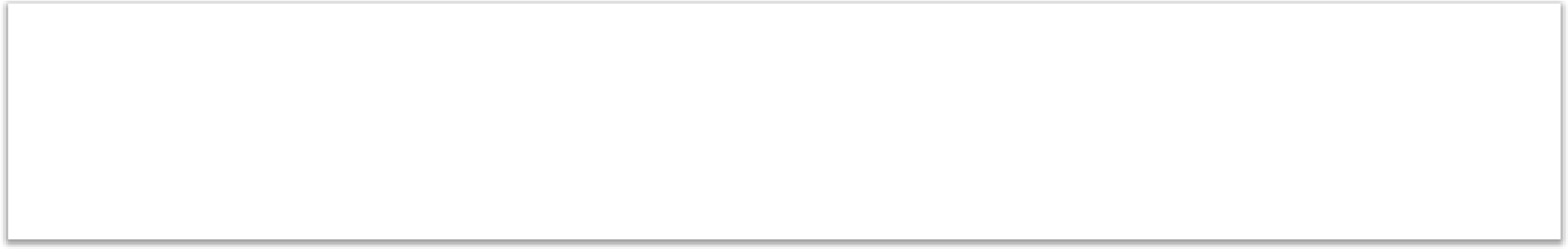


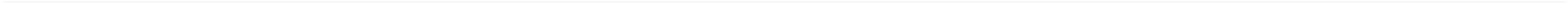
**How many hours per week do you spend directly with your horse or horses?**(eg. Grooming, riding, feeding etc)


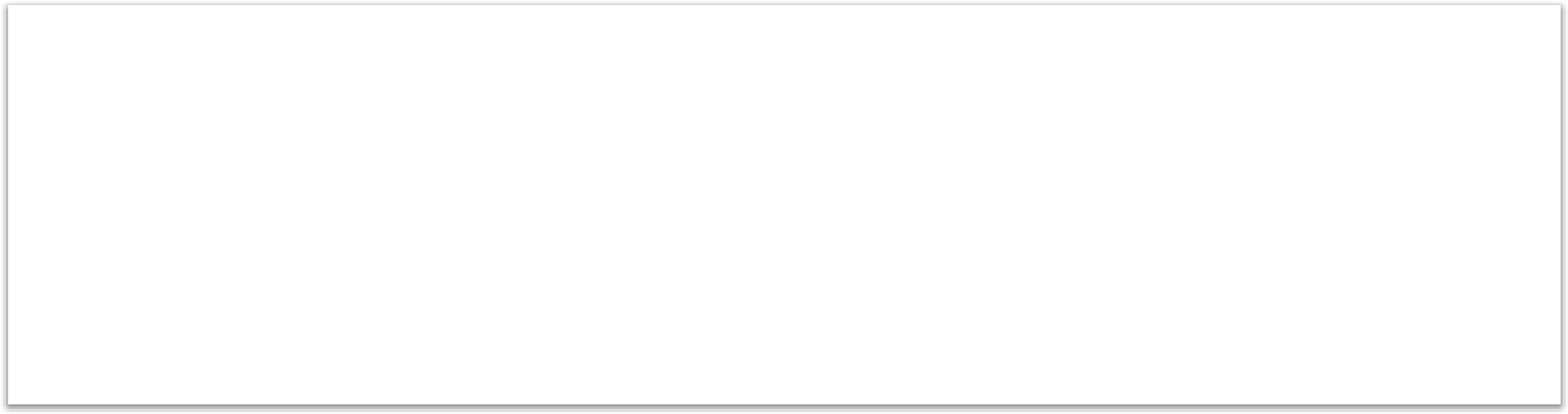


0

hours

5

Hours or less

6-15

hours

16-35

hours

35

Hours or more


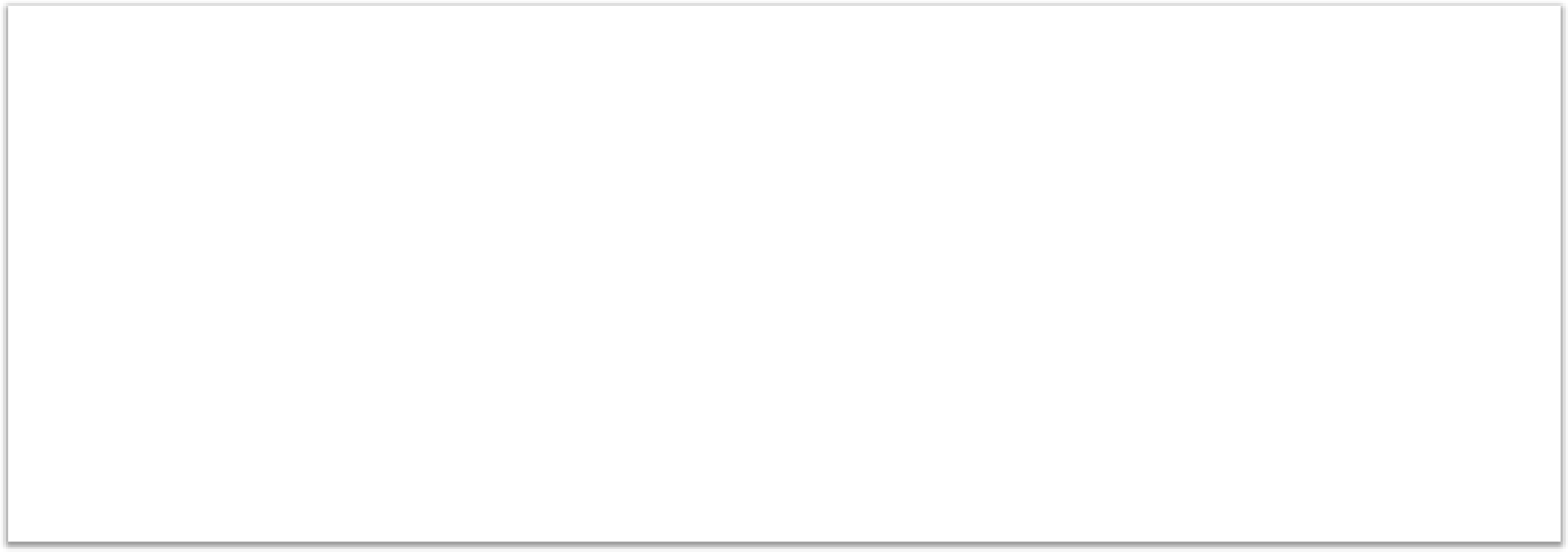


Yes, Personal liability

Yes, Loss of use

Yes, Surgical

Yes, Major medical

Yes, Limited mortality

Yes, Unsure of policy type

No

**Is your horse insured?**

**To what extent does your insurance status affect your interactions with veterinary support?**


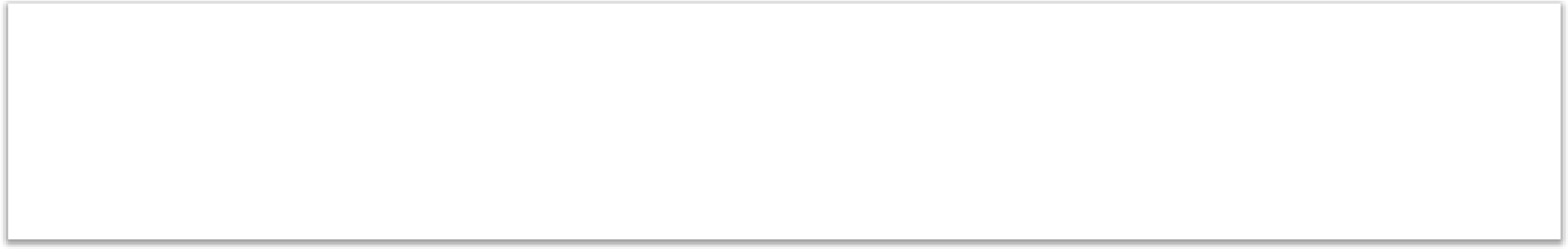


# **Antibiotic Usage**

**Where do you** **source** **your antibiotic treatments from?** (Please tick all that apply).

Please don't select more than 1 answer(s) per row.

|  | Never | Rarely | Sometimes | Often | Always |
| --- | --- | --- | --- | --- | --- |
| Fellow horse owner |  |  |  |  |  |
| I had some left over and reused these |  |  |  |  |  |
| Online without vet prescription |  |  |  |  |  |
| Online with vet prescription |  |  |  |  |  |
| Vet |  |  |  |  |  |
| Direct from vet |  |  |  |  |  |
| Other |  |  |  |  |  |


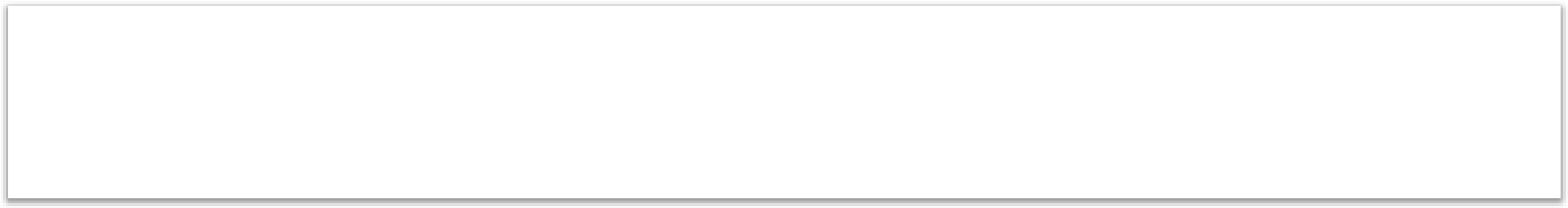


Yes

No

**Has your horse(s) had antibiotic treatment in the last two years?**

What was the antibiotic(s) for? Please list any details you recall per treatment.


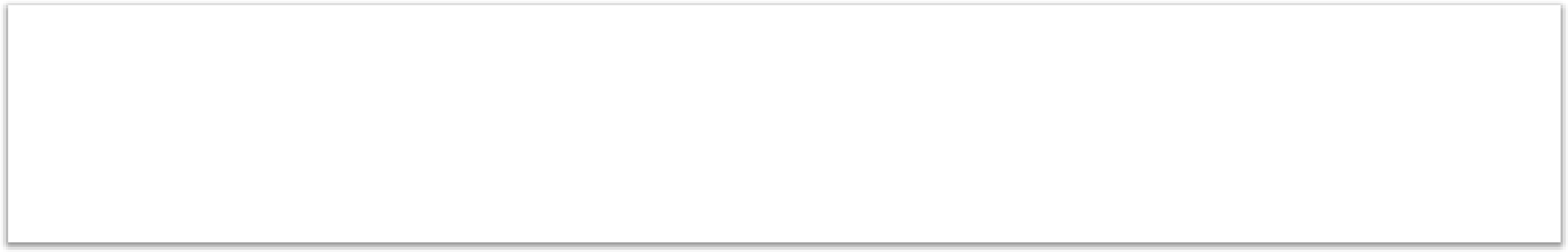


**How much would you agree with the following factors regarding antibiotic usage.**

Please don't select more than 1 answer(s) per row.

|  | Strongly Disagree | Disagree | Neither  Agree nor  Disagree | Agree | Strongly  Agree |
| --- | --- | --- | --- | --- | --- |
| Vet has advised therefore antibiotics are required |  |  |  |  |  |
| Vets should make treatment as cheap as possible |  |  |  |  |  |
| If my horse requires antibiotics, I want the vet to provide one clear recommendation that is the best option for my horse |  |  |  |  |  |
| If my horse requires antibiotics, I want the vet to provide several treatment options and let me decide which one is the best option |  |  |  |  |  |
| The ease of giving the antibiotic to my horse is an important factor to consider when discussing antibiotic usage (tablet, injection, in-feed) |  |  |  |  |  |
| I would probably choose a cheaper treatment option over a more expensive option |  |  |  |  |  |


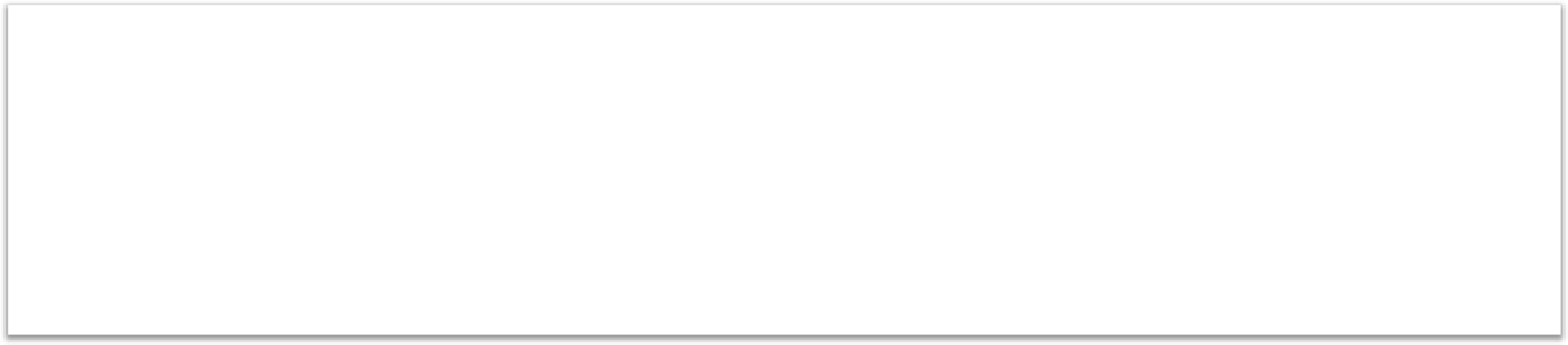


Yes, and I have asked for them specifically

Yes, and I have hinted that I wanted them, but I have never asked

Yes, but I have never hinted or asked

Never

**Have you ever wanted a vet to specifically give your horse(s) antibiotics?**

**Have you ever been surprised, disappointed or frustrated when a vet did not give your horse antibiotics?**


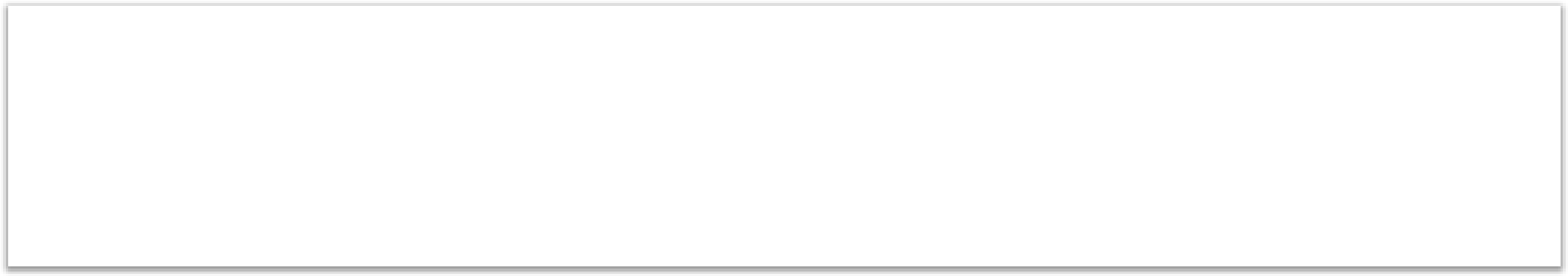


Yes,very

Yes,a little

No

**Would you ever consider changing to a different vet if your vet did not give you antibiotics you had asked for?**


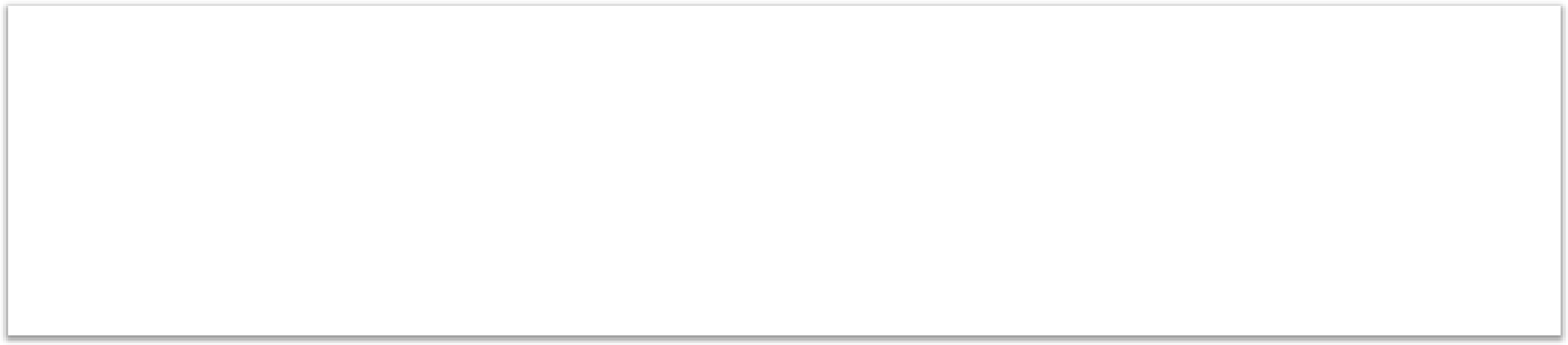


Yes

Possibly

No

N/a - I would not ask for antibiotics

**Would you ever consider sourcing antibiotics elsewhere (e.g. from a friend/yard manager/online) if your vet did not give you the medication you wanted?**


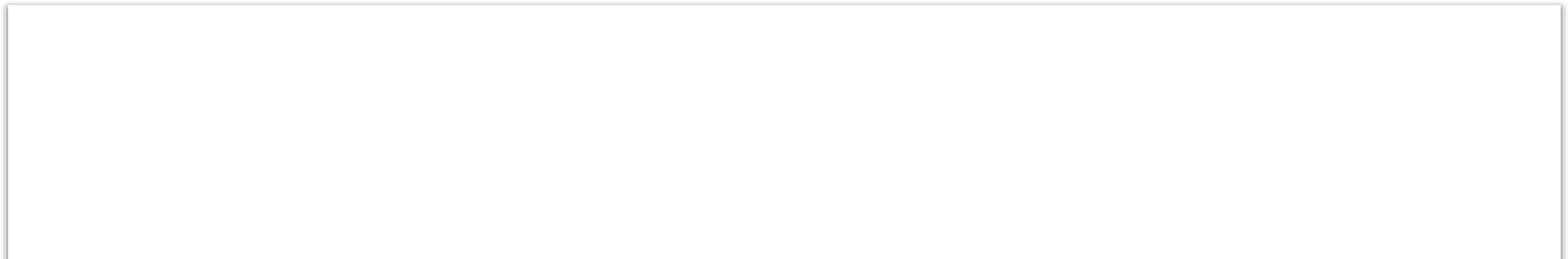


Yes

Possibly

No


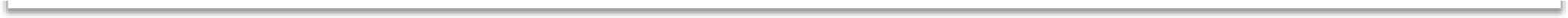


**How much do you agree with the following statements?**

Please don't select more than 1 answer(s) per row.

|  | Strongly Disagree | Disagree | Neither  Agree nor Disagree | Agree | Strongly  Agree |
| --- | --- | --- | --- | --- | --- |
| Antibiotics are required to treat all types of infections (viral or bacterial) |  |  |  |  |  |
| Vets should only prescribe antibiotics for treatment when they are required |  |  |  |  |  |
| Vets have a responsibility to protect both animal and human health |  |  |  |  |  |
| If antibiotics can be avoided, I would prefer my horse(s) not to receive them |  |  |  |  |  |
| Giving my horse(s) antibiotics can have a negative impact on their health |  |  |  |  |  |
| Antibiotics will ensure a sick horse gets better quicker |  |  |  |  |  |

| A course of antibiotics for my horse(s) should always be completed even if there is no longer any sign of infection/illness |  |  |  |  |  |
| --- | --- | --- | --- | --- | --- |

# **Antimicrobial Resistance**


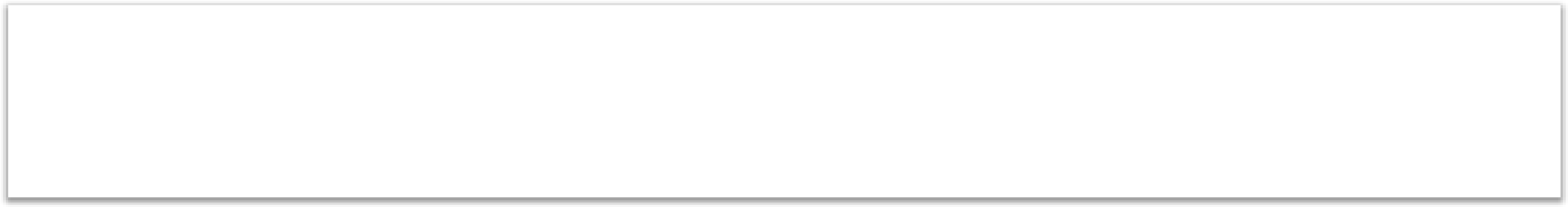


Yes

No

**Have you heard of antimicrobial resistance?**

**What does antimicrobial resistance mean to you?**

We are interested in any thoughts you are happy to share, whether brief or detailed.


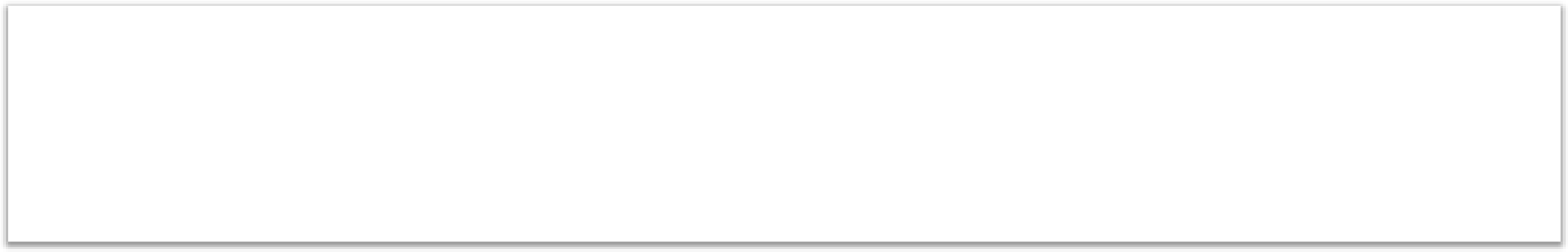


**Do you have anything else that might be important or useful, that you would like to say**


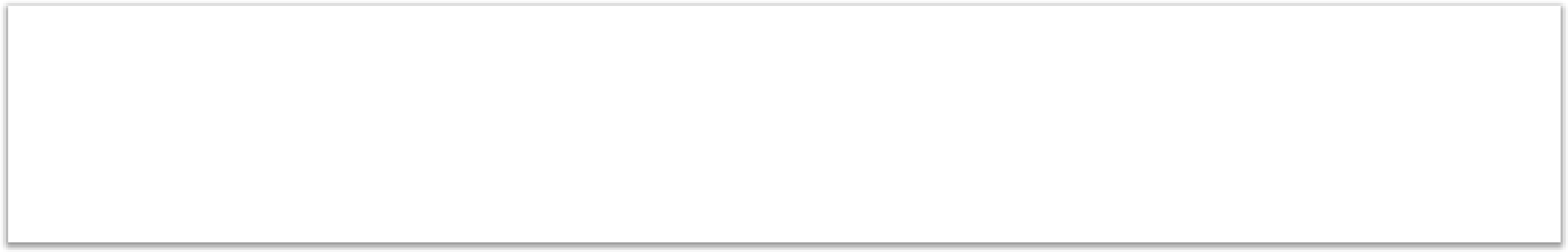

Supplement: Supplementary file 1 — Data S1. Survey S1: Equine survey. [file EVJ-58-564-s001.docx]
