# Supplementary material for: Understanding antimicrobial use by equine owners in Wales: Using cross‐sectional survey and semi‐structured interviews
Source: Equine Vet J. 2025 May 20;58(2):564–79. doi: 10.1111/evj.14522 (PMC12892391; doi:10.1111/evj.14522)
Supplement: Supplementary file 2 — Data S2. Schedule S2: Interview schedule. [file EVJ-58-564-s003.docx]

**Interview Schedule**

Demographic Information questions

Name

Age

Sex

Postcode

Role

**General Introduction: a bit about you**

- Could you tell me about your history with horses, personally and professionally: what got you started in the equestrian world? How long have you owned/looked after/worked with horses?
- How would you describe your *current* role/relationship to horse(s): ownership/shared ownership/stewardship/care for?
- How many horses do you currently [own/share/steward/care for]?
- Tell me about this horse/these horses: breed, age, purpose (e.g., pleasure, competition, breeding etc.)
- Tell me a little about the day-to-day human-animal interaction with this horse/these horse(s):
  - where are they kept
  - who is responsible for exercise/husbandry/health care decision making and activities?
- Can you tell me the positive and negative things about your current role/relationship with horses?

**Human-animal relationship and interaction**

- How would you describe you relationship with the horse(s) you [own/share/steward/care for]?
- How does this relationship compare to other human-animal relationships you’ve experienced? (e.g., farm, companion)

**Animal Health care**

- How would you describe the general health of the horse(s) you [own/share/steward/care for]?
- What measures do you put in place for ensuring horse health?
  - *E.g. biosecurity measures, diet, exercise, health reviews*
- How do you assess the health of your horse(s)?
- If you notice your horse(s) are unwell, who do you turn to first for advice?
- [Get them to expand on this **if not the vet**, if the vet move on to next]

**Veterinary relationship**

- How would you describe your relationship with your vet?
- How often do you see your vet in a given year?
- What kind of things would give you a cause to call your vet?
- Could you describe for me the veterinarian’s role with the horse(s) you [own/share/steward/care for]?
  - - *Just a conduit for medicine, or mostly for advice?*
    - *Health planning or emergency treatment*
    - *One vet or many?*

**Treatment decision making [i.e., antimicrobials]**

- To what extent do you feel you have an active role in treatment decision making?
- What factors come into play when considering treatment options available to you?
  - *E.g., cost, animal age, animal welfare, animal health, administration ease etc.*
- What information does the vet provide about treatments?
  - (e.g. think back to the last prescription made)
    - Do you find this information useful?
    - Is it clear? Easy to understand and utilise?
    - How would you prefer to get this information about the medicines?
- If you think back to the past three times your horse has had AMs prescribed, how easy was it for you and any other carers of your horse(s) to complete the course duration and administration as prescribed by your vet?
- If it is not completed, what happens with the remaining medication?
- If it is stored, is it something that you and any other carers of your horse(s) would ever make use of?
  - Could you give me an example if this has happened in the past?
- What other factors are important in discussions/veterinary advice on treatment?
  - What makes veterinary advice in this area more or less useful and/or feasible?

**Anti-Microbial Resistance**

- Have you heard of Anti-Microbial resistance?
  - If yes, ask for their understanding.
- Does this understanding influence decisions in relation to your own animal(s)?
- Do you have/Is there an AMU/AMR policy/ guidelines within your stable?/who do you ask if you are unsure? (Policy for Livery managers, Livery stabled horse owners and small holdings, guidelines for independent owners).
- In the equine context, what do you feel might be the biggest challenges surrounding better or more responsible AMU?

**To finish: Do you have anything else that might be important or useful, that you would like to say?**
