# Supplementary material for: Understanding antimicrobial use by equine owners in Wales: Using cross‐sectional survey and semi‐structured interviews
Source: Equine Vet J. 2025 May 20;58(2):564–79. doi: 10.1111/evj.14522 (PMC12892391; doi:10.1111/evj.14522)
Supplement: Supplementary file 3 — Table S1. Interview participant information including roles they fulfilled. [file EVJ-58-564-s002.pdf]

Table S1: Interview participant information including roles they fulfilled.

Description of interviewee	Age Range	Sex	Activity(s) they were involved in	Role(s) they fulfilled
**1**	35-44	Female	Leisure/pleasure	Owner
**4**	35-44	Female	Wide Range	Yard manager, Owner, Rider
**5**	35-44	Female	Competitive, Leisure/pleasure	Livery yard owner, Owner, Rider
**6**	55-64	Female	Leisure/pleasure	Owner, Rider, Pony Club Official
**8**	35-44	Female	Leisure/pleasure	Owner, Rider
**7**	55-64	Female	Competitive, Leisure/pleasure	Owner, Rider
**9**	75+	Female	Leisure/pleasure	Horse Owner
**10**	55-64	Female	Leisure/pleasure	Owner, Rider, Retired Equine Lecturer
**15**	65-74	Female	Competitive, Leisure/pleasure	Horse owner/Endurance Rider
**17**	35-44	Female	Leisure/pleasure	Owner, Rider
**19**	55-64	Female	Leisure/pleasure	Owner, Rider
**20**	55-64	Female	Wide Range	Owner, Rider, Breeder, Trainer, Judge
**21**	55-64	Female	Competitive Dressage, Pony Club	Owner, Rider
**22**	55-64	Female	Leisure/pleasure	Owner, Rider
**23**	75+	Female	TREC	Horse owner/rider
**24**	55-64	Female	Competitive, Dressage	Horse owner/Endurance Rider
**25**	65-74	Female	Leisure/pleasure	Livery yard owner, Owner, Rider
**26**	55-64	Female	Wide Range	Livery yard owner, Owner, Rider, Breeder
**27**	55-64	Female	Leisure/pleasure	Owner, Rider
**28**	55-64	Female	Leisure/pleasure	Owner, Rider
**29**	65-74	Male	Leisure/pleasure	Owner, Rider
